# Linking CREB function with altered metabolism in murine fibroblast-based model cell lines

**DOI:** 10.18632/oncotarget.22135

**Published:** 2017-10-27

**Authors:** André Steven, Sandra Leisz, Claudia Wickenhauser, Kristin Schulz, Dimitrios Mougiakakos, Rolf Kiessling, Carsten Denkert, Barbara Seliger

**Affiliations:** ^1^ Institute of Medical Immunology, Martin Luther University Halle-Wittenberg, Halle, Germany; ^2^ Institute of Pathology, Martin Luther University Halle-Wittenberg, Halle, Germany; ^3^ Department of Internal Medicine 5, Hematology and Oncology, University of Erlangen-Nuremberg, Erlangen, Germany; ^4^ Karolinska Institute, CCK, Stockholm, Sweden; ^5^ Charité Berlin, Institute of Pathology, Berlin, Germany

**Keywords:** CREB, HER-2/neu, metabolism, mitochondria, ROS

## Abstract

The cAMP-responsive element binding protein CREB is frequently overexpressed and activated in tumors of distinct histology, leading to enhanced proliferation, migration, invasion and angiogenesis as well as reduced apoptosis. The de-regulated expression of CREB might be linked with transcriptional as well as post-transcriptional regulation mechanisms. We show here that altered CREB expression levels and function are associated with changes in the cellular metabolism. Using comparative proteome-based analysis an altered expression pattern of proteins involved in the cellular metabolism in particular in glycolysis was found upon CREB down-regulation in HER-2/neu-transfected cell lines. This was associated with diminished expression levels of the glucose transporter 1, reduced glucose uptake and reduced glycolytic activity in HER-2/neu-transfected cells with down-regulated CREB when compared to HER-2/neu^+^ cells. Furthermore, hypoxia-induced CREB activity resulted in changes of the metabolism in HER-2/neu transfected cells. Low pH values in the supernatant of HER-2/neu transformants were restored by CREB down-regulation, but further decreased by hypoxia. The altered intracellular pH values were associated with a distinct expression of lactate dehydrogenase, and its substrate lactate. Moreover, enhanced phosphorylation of CREB on residue Ser133 was accompanied by a down-regulation of pERK and an up-regulation of pAKT. CREB promotes the detoxification of ROS by catalase, therefore protecting the mitochondrial activity under oxidative stress. These data suggest that there might exists a link between CREB function and the altered metabolism in HER-2/neu-transformed cells. Thus, targeting these altered metabolic pathways might represent an attractive therapeutic approach at least for the treatment of patients with HER-2/neu overexpressing tumors.

## INTRODUCTION

The 43 kDa cyclic AMP (cAMP)-responsive element binding protein (CREB) is a member of the large family of basic leucine zipper (bZIP)-containing transcription factors (TF) and plays a key role in many physiologic as well as pathophysiologic processes including cell growth, differentiation, apoptosis, cell cycle progression and immune response amongst others [1]. CREB exerts distinct activities in context and time-dependent manners [2–4], which depend on its phosphorylation status mediated by various kinases. Phosphorylated CREB interacts with the CREB-binding protein CBP/p300 via its kinase inducible domain (KID) and the KID-interacting domain (KIX) in CBP/p300 [5]. The CREB/CBP complex induces a transcriptional machinery leading to activation of cAMP-responsive element (CRE) containing gene promoters. CREB mediates transcriptional responses to different stimuli including hormones, growth factors and neurotransmitters thereby acting as mediator between signaling pathways and their downstream activation target [3, 6].

However, based on its frequent overexpression and persistent activation CREB is also involved in the malignant transformation process [7]. This is mediated by aberrant activation of cAMP signal transduction-dependent pathways such as G-coupled receptors, receptor tyrosine kinase (RTK) like human epidermal growth factor receptor 2 (HER-2/neu) and the cytokine/JAK/STAT signaling cascades, as well as signaling events downstream of CREB.

HER-2/neu is a member of the transmembrane epidermal growth factor receptor (EGF-R) family and is physiological expressed in many epithelial cells. However, it is overexpressed and/or amplified in human tumor of distinct origin including mammary carcinoma [8] and colorectal cancer [9]. HER-2/neu transformation is associated with an increased cell proliferation, migration, enhanced cell cycle progression as well as a survival of patients [10]. Recently, we could demonstrate in the murine model system used also in this study, that HER-2/neu can increase the phosphorylation rate of CREB and that HER-2/neu overexpressing cells had more detectable CREB protein than the parental NIH3T3 cells [11].

As recently reviewed CREB overexpression and its constitutive activation was found in many solid tumors, including non-small cell lung carcinoma, glioblastoma, melanoma, mammary carcinoma, renal cell carcinoma and mesothelioma as well as in different hematopoietic malignancies [4, 12–18], leading to enhanced cell proliferation, reduced apoptosis, increased angiogenesis and migration rates. Downregulation of CREB expression inhibits proliferation, migration and/or invasion, which was accompanied by suppression of matrix metallopeptidases and proteins of the epithelial mesenchymal transition [18]. Furthermore, CREB overexpression can be correlated with clinico-pathological parameters, in particular with tumor grade, stage, metastasis formation, poor prognosis and reduced patients’ survival [14, 19–22]. In addition, CREB overexpression is often caused by an up-regulation of downstream target genes involved in neoplastic transformation as demonstrated by different high throughput analysis [23].

The expression level and activity of CREB is dynamically regulated by different mechanisms [24] and critical for maintaining a homeostatic cellular environment [25]. CREB transcriptionally controls a large number of putative target genes, which are involved in numerous cellular processes, such as signal transduction, cell structure, differentiation, cell survival as well as proliferation [17]. So far, amplifications and/or deletions in CREB have only been rarely detected suggesting that rather deregulation mechanisms of CREB appear to be responsible for its overexpression in tumors [26, 27]. Recently, a post-transcriptional control mechanism for CREB has been described, which seems to be mediated by microRNAs (miRs) known to for their binding to the 3' untranslated region (UTR) of CREB thereby contributing to tumorigenesis in different tumor models as demonstrated both *in vitro* and *in vivo* [28–31]. In addition, there is increasing evidence that different extra-cellular signals have an impact on the tumor microenvironment (TME), like hypoxia, pH variation and oxidative stress [32]. Furthermore, post-translational modifications (PTM) of CREB, which can be quite diverse including phosphorylation, ubiquitination, methylation, glycosylation and SUMOylation, might have an impact on CREB function(s) [3, 17, 33]. So far, a link between CREB expression levels/function(s) and tumor metabolism has not been identified. Therefore, this study analyzed the effects of CREB on the metabolism using a murine model of HER-2/neu transformation with distinct CREB expression and activation levels, which has been previously well characterized and was able to induce tumors in immunocompetend DBA mice [11, 17, 34].

## RESULTS

### CREB-mediated changes in the protein expression pattern

**Figure 1:**
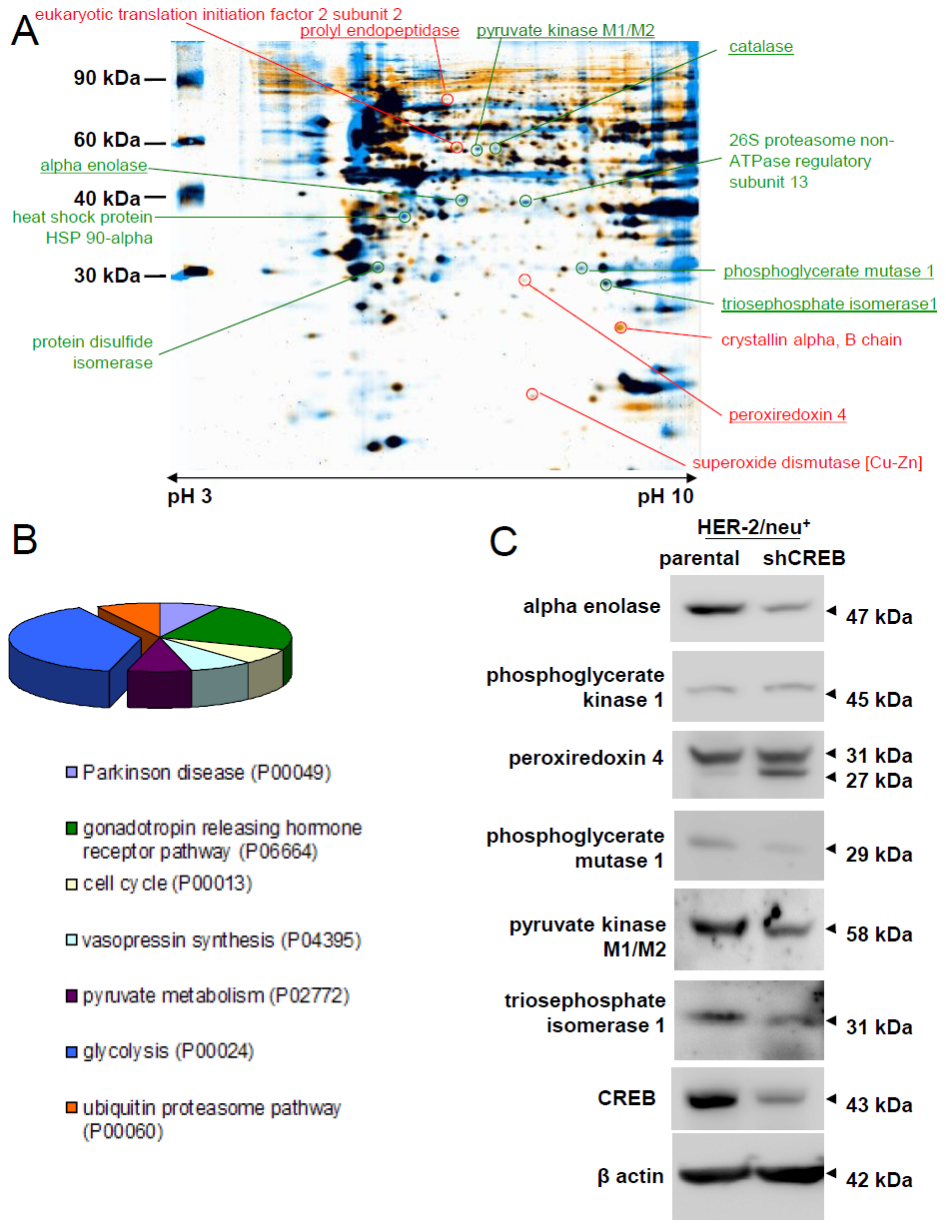
Influence of CREB knock down on metabolism and glycolytic enzymes **(A)** HER-2/neu^+^ and CREB-downregulated HER-2/neu^+^ (shCREB) cells were lysed and the protein pattern was analyzed by 2-DE gel electrophoresis following staining of the gels with colloidal Coomassie Blue. Differentially regulated protein spots of interest were digested with trypsin and the fragments were identified by MS/MS-TOF analysis. The picture represents a representative overlay of merged profiles (four technical replicates for both cell lines) in sum of three independent experiments. Some identified proteins are marked and listed. Red marked spots were up-regulated in CREB knock down cells, whereas green marked spots were down-regulated. The underlined proteins were further validated by measuring the mRNA expression status. **(B)** The graph shows the distribution pattern of the identified targets in (A) based on their classification into various pathways, functional roles or diseases and was performed by using the PANTHER database (pantherdb.org). **(C)** Validation of the differentially expressed proteins in (A). The representative protein expression pattern in one HER-2/neu overexpressing cell line (HER-2/neu^+^, left lane) and one shCREB HER-2/neu^+^ (right lane) is shown for metabolic enzymes. The blot represents one of three experiments.

Since the level of CREB and HER-2/neu expression has been correlated with growth characteristics and altered signaling cascades [32], the protein expression pattern of HER-2/neu^+^ versus CREB-diminished HER-2/neu^+^ (shCREB) cells (Supplementary Figure 1A), with a knock down of up to 80% on the protein level (Supplementary Figure 1B, 1C) were determined by using two-dimensional gel electrophoresis (2-DE)-based proteome analysis and differentially expressed protein spots, defined by a 2-fold regulation, were identified by mass spectrometry. Overall 23 differentially expressed protein spots have been identified from three biological replicates (merged gels of all three experiments can be found in Supplementary Figure 2A), from which 13 proteins were down-regulated including four different forms of alpha-tubulin and 10 up-regulated upon CREB down-regulation. The differentially expressed proteins were mainly involved in metabolic processes (Table 1, Figure 1A, Supplementary Figure 2B), in particular in glycolysis (Figure 1B). Based on their distinct expression pattern the following candidate CREB-regulated proteins were selected and their expression validated by qPCR and/or Western blot analyses: The panel of potential targets includes the phosphoglycerate kinase (PKG)1, prolyl endopeptidase, peroxiredoxin (PRX)4, enolase (ENO), triose phosphate isomerase (TPI), pyruvate kinase M (PKM) and citrate synthase. In line with the proteomic profiling data reduced transcription levels of PKM, citrate synthase and TPI were found in CREB down-regulated HER-2/neu^+^ cells (Table 2), while the mRNA expression level of PGK1 remained unchanged and that of the prolyl endopeptidase (PEP) induced. In addition, a CREB-mediated transcriptional control was detected for cofilin and α-crystalline (Table 2). The decreased mRNA levels were associated with decreased protein expression levels of ENO, PRX4, PGK1, PGAM1, PKM and TPI in HER-2/neu^+^ shCREB versus HER-2/neu^+^ cells (Figure 1C), which was further confirmed by a down-regulated PKM, TPI, and PGK1 enzyme activity (Table 3). Other differentially expressed proteins were enzymes important for detoxification mechanisms (catalase, PRX4, superoxide dismutase [Cu-Zn]) or linked to the protein degradation process (26S proteasome non-ATPase regulatory subunit 13, PEP, leukocyte elastase inhibitor A) (Table 1).

**Table 1:** CREB-regulated proteins identified by 2-DE-based proteomics

Protein	Acc no.	Molecular weight estimated found		IP	Ratio (range)	Sequence coverage	Score (range)	Localization	Function
pyruvate kinase isozymes M1/M2	P52480	58.3	55	7.18	0.09 - 0.201	19	78 - 212	cytoplasm and nucleus	glycolysis, degradation of carbohydrates
heat shock protein HSP 90-alpha	P07901	83.5	**38**	4.93	0.145 - 0.192	14	56 - 62	cytoplasm and melanosome	chaperon, cell cycle control, signal transduction
tubulin alpha-1A chain	P68369	50.7	45	4.94	0.179 - 0.45	25	83 - 102	cytoplasm and cytoskeleton	microtubules
tubulin alpha-1B chain	P05213	50.8		4.94		25	83 - 102		
tubulin alpha-1C chain	P68373	50.5		4.96		26	83 - 102		
tubulin alpha-3 chain	P68373	50.6		4.97		20	53 - 74		
phosphoglycerate kinase 1	P09411	44.9	41	8.02	0.228 - 0.457	38	66 - 116	cytoplasm	glycolysis, degradation of carbohydrates, angiogenesis
catalase	P24270	59.7	57	7.72	0.248 - 0.324	29	76 - 90	peroxisome	detoxification of hydrogen peroxide
alpha-enolase	P17182	47.5	41	6.37	0.125 - 0.448	35	116 - 128	cytoplasm and cell membrane	glycolysis, growth, regulation of hypoxic events
phosphoglycerate mutase 1	Q9DBJ1	28.9	30	6.67	0.077 - 0.392	31	101 - 135	cytoplasm and membrane	glycolysis
vimentin	P20152	53.7	60	5.06	0.244 - 0.33	29	62 - 81	cytoplasm	structural protein
protein disulfide-isomerase A6	P09103	48.5	42	5.00	0.273 - 0.46	21	78 - 80	ER lumen	yield of disulfide bridges, chaperone
triosephosphate isomerase	P17751	32.6	29	5.56	0.246 - 0.483	33	92 - 191	nucleus	glycolysis, gluconeogenesis
prolyl endopeptidase	Q8C167	81.6	77	5.44	2.144 - 3.569	20	136 - 202	cytoplasm	serine protease, synthesis and degradation of peptide hormones
spliceosome RNA helicase Ddx39b	Q9Z1N5	49.4	50	5.44	2.01 - 4.405	35	61 - 95	nucleus	splicing
26S proteasome non-ATPase regulatory subunit 13	Q9WVJ2	43.1	39	5.46	2.352 - 2.725	57	65 - 245	cytoplasm	protein degradation, regulation of proteasomal activity
superoxide dismutase [Cu-Zn]	P08228	16.1	23	6.02	2.574 - 3.33	38	80 - 91	cytoplasm	radical detoxification
flavin reductase (NADPH)	Q923D2	22.2	24	6.49	2.696 - 3.244	38	84 - 104	cytoplasm	oxidoreductase
peroxiredoxin-4	O08807	31.2	28	6.67	2.704 - 5.096	40	59 - 98	cytoplasm and extracellular	redox regulation, regulation of NF-k-B activity
ATP-dependent RNA helicase DDX39A	Q8VDW0	49.5	60	5.46	3.473 - 4.461	43	167 - 188	cytoplasm and nucleus	splicing
leukocyte elastase inhibitor A	Q9D154	42.7	40	5.85	7.577 - 9.137	33	138 - 183	cytoplasm	regulation of protease activity
cofilin-1	P18760	18.7	21	8.22	21.579 - 24.771	46	92 - 107	cytoplasm and nucleus	cell morphology and cytoskeleton, mitosis
alpha-crystallin B chain	P23927	20.0	22	6.76	20.082 - 86.972	58	108 - 173	cytoplasm and nucleus	stress induced chaperone, protection against protein aggregation

Functions, localization, IP, estimated molecular weigth and accession number of the proteins were added according to the MASCOT and SwissProt database.

Up-regulated proteins after CREB knock down are marked in red, down-regulated proteins in green. The red bold value in the row “molecular weight found” indicates samples with an alteration of the molecular weight by 15 or more percent compared to the estimated molecular weight. For the ratio and the score value the range of the three experiments is given.

**Table 2:** mRNA expression level of metabolic and stress related genes as identified to be differentially expressed in 2D gel electrophoresis of CREB down-regulated vs. HER-2/neu^+^ and NIH3T3 cells

	NIH3T3	NIH3T3 shCREB	HER-2/neu^+^	HER-2/neu^+^ shCREB
citrate synthase	1	0.87 +/- 0.28	1.09 +/- 0.13	0.34 +/- 0.05 ^*^
cofilin 1	1	0.68 +/- 0.12 ^*^	0.41 +/- 0.16 ^*^	0.93 +/- 0.22 ^*^
α-crystalline B	1	0.61 +/- 0.17 ^*^	0.45 +/- 0.12 ^*^	0.2 +/- 0.04 ^*^
peroxiredoxin 4	1	1.23 +/- 0.42	0.81 +/- 0.17	1.76 +/- 0.18 ^*^
PGAM1	1	0.89 +/- 0.17	1.87 +/- 0.12 ^*^	1.21 +/- 0.2 ^*^
PGK1	1	0.68 +/- 0.18 ^*^	1.76 +/- 0.45 ^*^	0.78 +/- 0.02 ^*^
prolyl endopeptidase	1	0.42 +/- 0.12 ^*^	0.3 +/- 0.08 ^*^	0.57 +/- 0.08 ^*^
pyruvate kinase M1/2	1	0.62 +/- 0.05 ^*^	1.68 +/- 0.14 ^*^	0.12 +/- 0.03 ^*^
triose phosphate isomerase	1	0.7 +/- 0.12 ^*^	4.97 +/- 1.11 ^*^	4.82 +/- 1.23

Expression levels of the parental NIH3T3 were normalized to 1. For the significance of the row HER-2/neu^+^ the values were compared to that of the parental NIH3T3.

The expression of the genes analysed was compared to parental NIH3T3 cells (normalizied to 1). * statistical significant (p < 0.05).

**Table 3:** Modulation of enzymatic activities by CREB down-regulation in HER-2/neu^+^ cells

Activity [U/mg total protein]	HER-2/neu^+^	HER-2/neu^+^ shCREB
PKM	12.64 + 0.52	3.01 +/- 0.7 ^*^
TPI	38.64 +/- 0.31	6.81 +/- 0.47 ^*^
PGAM1	5.24 +/- 0.61	2.28 +/- 0.24 ^*^
PGK	5.45 +/- 0.62	2.56 +/- 0.21 ^*^
prolyl endopeptidase	0.63 +/- 0.45	1.57 +/- 0.54
peroxiredoxin 4	1.48 +/- 0.79	3.44 +/- 0.35 ^*^

The enzyme activity was calculated to the protein concentration.

*In silico* analysis of gene promoters from differentially expressed proteins upon CREB down-regulation revealed that most of the identified proteins were controlled by half CRE sites (TGACG or CGTCA), whereas full CRE sites (TGACGTCA) were merely found in promoters of up-regulated proteins (Tables 4 and 5). Since the promoter of the oncogene HER-2/neu contains no CRE elements, its expression was not affected by CREB down-regulation [11].

**Table 4:** CRE elements in gene promoter of differential regulated proteins identified after CREB knock down by 2-DE and MS

Symbol	Gene name	Accession	CRE prediction	CRE flag	Full site	Half site	Conserved CRE	CRE cluster	pHMM+Pos	Chrom. localization	Strand	Pos
Pkm2	“pyruvate kinase, muscle”	NM_011099	Others	ht h		ht_-1433 h_-2				chr9	+	59830243
Pkm2	“pyruvate kinase, muscle”	NM_011099	Others	none		h_506 h_630				chr9	+	59830243
Hspca	“heat shock protein 1, alpha”	NM_010480	Others	h		ht_-4769 ht_-3435 h_0 ht_830 h_893				chr12	-	104744737
Tuba1	“tubulin, alpha 1”	NM_011653	Others	ht		ht_-2171				chr15	-	99318440
Tuba2	“tubulin, alpha 2”	NM_011654	Others	none						chr15	-	99299381
Tuba6	“tubulin, alpha 6”	NM_009448	Others	h		ht_-3468 h_-497 h_18 ht_575				chr15	+	99396317
Tuba7	“tubulin, alpha 7”	NM_009449	Others	ht		ht_95				chrUn_random	+	117747169
Pgk1	phosphoglycerate kinase 1	NM_008828	Others	ht		ht_-1021 ht_-707 ht_512				chrX	+	91115110
Cat	catalase	NM_009804	Others	ht		h_-4839 ht_-2392 h_910				chr2	-	104935582
Eno1	“enolase 1, alpha non-neuron”	NM_023119	Others	none		h_527				chr4	+	146873883
Pgam1	phosphoglycerate mutase 1	NM_023418	Others	none		ht_-4353 ht_-3676				chr19	+	41498057
Vim	vimentin	NM_011701	others	ht h		ht_-580 h_-226				chr2	+	13567777
Tpi	triose phosphate isomerase	NM_009415	others	none		h_-3599				chr6	-	125622026
Prep	prolyl endopeptidase	NM_011156	others	ht		ht_-4229 ht_-918 ht_613				chr10	+	44917660
Bat1a	HLA-B-associated transcript 1A	NM_019693	CRE_TATA	none		ht_-4510 ht_-3773 ht_-3526 h_886			FF_human_1.0_-4904_mouse_1.3_-5083	chr17	+	33858338
Psmd13	“proteasome (prosome, macropain) 26S subunit, non-ATPase, 13”	NM_011875	others	ht h		h_-4162 ht_-1835 h_-65 ht_866				chr7	+	129902896
Blvrb	biliverdin reductase B (flavin reductase (NADPH))	NM_144923	CRE_NoTATA	H h		ht_-4463 H_-1237 h_-1191	H_-1237_635	|H_-1237_h_-1191_0		chr7	+	19020950
Prdx4	peroxiredoxin 4	NM_016764	CRE_TATA	HT FH h	FH_-1215	HT_-2035 h_-101	FH_-1215_378 HT_-2035_177			chrX	-	135097408
Ddx39	DEAD (Asp-Glu-Ala-Asp) box polypeptide 39	NM_197982	CRE_TATA	ht		ht_-4185 ht_-669 ht_702			FH_human_2.2_-3409_mouse_1.7_-3491	chr8	+	82993770
NE	neutrophil elastase	NM_015779	others	none		h_-4246 h_505				chr10	+	79703208
Cfl1	“cofilin 1, non-muscle”	NM_007687	CRE_TATA	none		ht_-3170 ht_558			FF_human_1.2_-8876_mouse_1.2_-8835	chr19	-	5190328
Cfl1	“cofilin 1, non-muscle”	NM_007687	CRE_TATA	none					FF_human_1.2_-8876_mouse_1.2_-8835	chr8	+	41275610
Cryab	“crystallin, alpha B”	NM_009964	others	ht h		ht_-4617 ht_-3003 ht_-1195 h_-716				chr9	+	50875865

Chr: chromosome.

**Table 5:** CRE elements in gene promoter of differential regulated proteins identified after CREB knock down by qPCR

Symbol	Gene name	Accession	CRE prediction	CRE flag	full site	Half site	Conserved CRE	CRE cluster	pHMM+Pos	Chrom localization	Strand	Pos
ERBB2 ^*^ (HER-2;HER2;NEU; NGL;TKR1)	“v-erb-b2 erythroblastic leukemia viral oncogene homolog 2, neuro/glioblastoma derived oncogene homolog (avian)”	NM_004448	others	none						chr17	+	38231358
Cs	citrate synthase	NM_026444	others	ht		ht_-2966 ht_-2751				chr10	+	128362478
Es10	esterase 10	NM_016903	others	ht h		h_-2215 ht_151 ht_481				chr14	+	65179735
Slc2a1 (Glut-1)	“solute carrier family 2 (facilitated glucose transporter), member 1”	NM_011400	CRE_NoTATA	ht h		ht_-3367 ht_-1283 h_62 h_91 h_560		|h_62_h_91_0		chr4	+	117243469
Gss	glutathione synthetase	NM_008180	others	ht h		ht_-2831 h_-2156 h_119 h_732				chr2	-	157439425

* - Erbb2 expression is unregulated after CREB knock down.

Chr: chromosome.

### Association of CREB and hypoxia-induced changes on the cell metabolism of HER-2/neu^+^ cells

**Figure 2:**
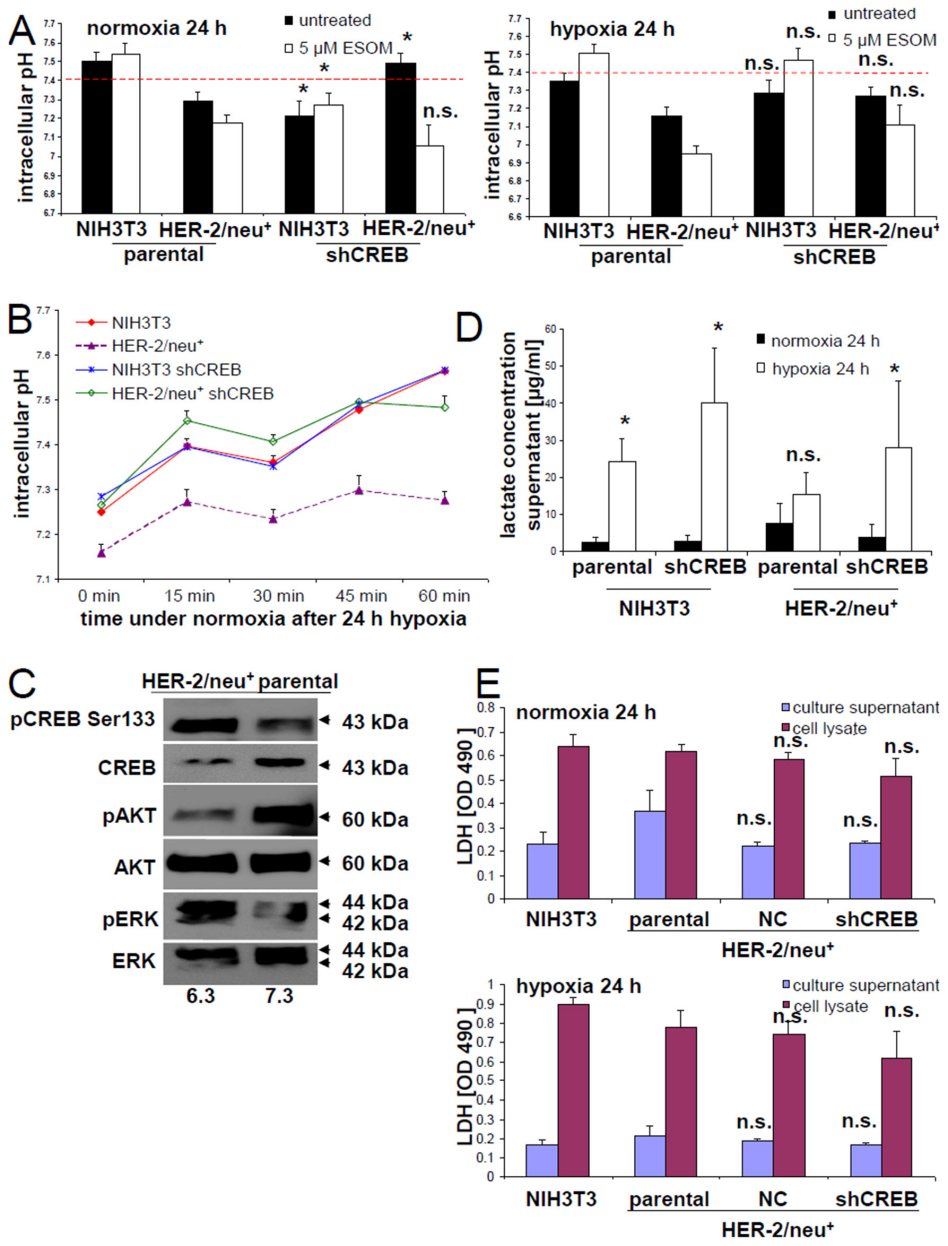
Induction of an altered microenvironment in CREB-reduced cells under hypoxia **(A)** Intracellular pH was determined by staining with BCECF-AM under the conditions given in Material and Methods. As a control, cells were treated with the inhibitor ESOM (5 μM/24 h) was used blocking the H^+^/K^+^ pump. **(B)** The intracellular pH was followed during the re-oxygenation phase after cultivation under hypoxic conditions for 24 h. The data represents one of two independently performed experiments. **(C)** The effects of distinct pH conditions in the culture medium on the phosphorylation of CREB, AKT and ERK was determined by Western blot. The blot represents one of two experiments. **(D)** Lactate levels in the culture supernatants of NIH3T3 (HER-2/neu^-^) vs. HER-2/neu^+^ cells were determined using a commercial kit. Data in the columns represent the average of three different experiments along with the error bars. **(E)** LDH activity in the supernatant and in the cells was measured with a cytotoxicity assay as described in Materials and Methods. The columns and bars represent the data from three experiments.

The comparative proteomics profiling of HER-2/neu^+^ cells and its shCREB derivatives suggests a link between the CREB expression levels/activation status and metabolic changes. In order to determine the functional relevance of this altered expression pattern, intracellular pH values were determined in the model system. Reduced intracellular pH values were detected in HER-2/neu^+^ cells when compared to NIH3T3 cells, while the intracellular pH values were restored to that of NIH3T3 cells by CREB down-regulation in HER-2/neu^+^ cells (Figure 2A). As expected treatment of the cells with the proton pump inhibitor Esomeprazole (ESOM), which served as control, strongly decreased the intracellular pH value in CREB down-regulated HER-2/neu^+^ cells (Figure 2A).

Although a correlation between hypoxic conditions and metabolic reprogramming as well as the survival of tumor cells has been demonstrated [35–37], the role of CREB in this process has not yet been analyzed in detail. Therefore, the effect(s) of hypoxia on the energy metabolism of HER-2/neu^+^ and HER-2/neu^+^ shCREB as well as HER-2/neu^-^ (NIH3T3) cells was compared by determining the intracellular and extracellular pH values, lactate and lactate dehydrogenase (LDH) concentrations. Under hypoxic conditions the intracellular pH was even further reduced in HER-2/neu^+^ cells, but also reduced in CREB down-regulated HER-2/neu^+^ cells (Figure 2A). The hypoxia-mediated decline of the intracellular pH values in HER-2/neu^+^ cells could be restored over time by culturing these cells under normoxia suggesting that this is a rather reversible process (Figure 2B).

Altered extracellular pH conditions also affected both CREB activity and CREB-relevant signaling pathways as determined by Western blot analysis of HER-2/neu^+^ cells cultured at pH 6.3 and pH 7.3, respectively (Figure 2C). A shift from slightly acidic towards neutral pH conditions resulted in a decreased pCREB^Ser133^ phosphorylation rate in HER-2/neu^+^ cells, which was accompanied by a down-regulation of pERK and an up-regulation of pAkt as well as of total CREB.

The modulation of the intracellular pH values was linked to changes of the LDH expression levels and of lactate concentrations as determined in the supernatants of normoxic and hypoxic HER-2/neu^+^ and CREB down-regulated HER-2/neu^+^ cells: The lactate concentrations were increased in HER-2/neu^+^ vs. HER-2/neu^+^ shCREB and NIH3T3 cells, while hypoxia enhanced lactate concentrations in all cell supernatants tested with the highest increase in supernatants of NIH3T3 cells (Figure 2D). Total LDH concentrations were comparable across all cells under normoxic conditions. Furthermore, culturing the cells under hypoxic conditions caused an up-regulation of total LDH activity in all cell lines to a similar extent (Figure 2E), while CREB knock down had no significant influence.

### HIF-1α inhibitor blocks CREB activity and regulates metabolism

**Figure 3:**
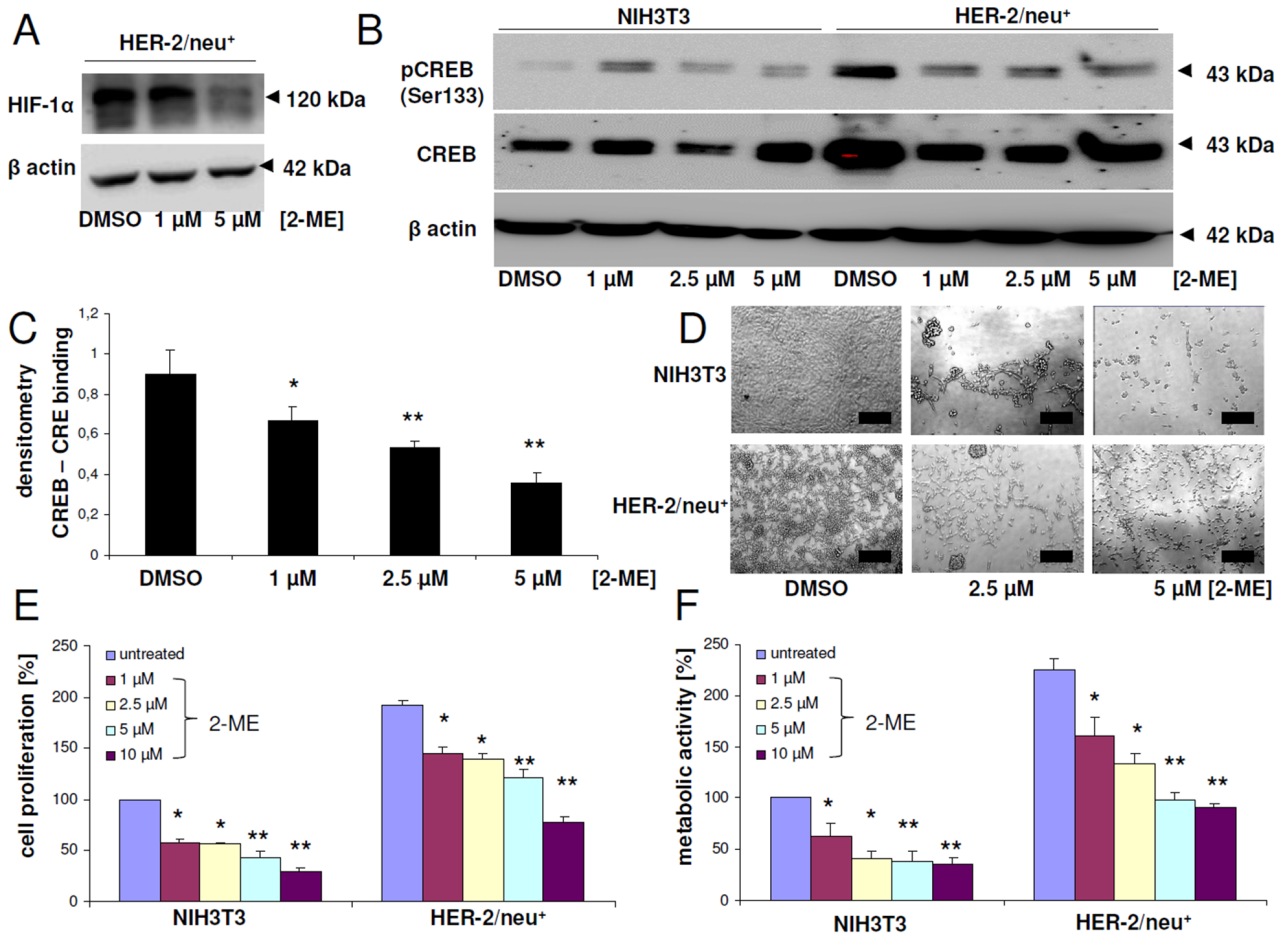
Modulation of CREB activity, proliferation and metabolic activity upon treatment with the HIF-1α inhibitor 2-methoxyestradiol **(A)** HER-2/neu^+^ cells were treated with 2-ME or with DMSO as the solvent control for 24 h with the indicated concentrations and HIF-1α expression was analyzed by Western blot. β actin served as a loading control. The bands represent one of three independent experiments. **(B)** NIH3T3 cells and HER-2/neu^+^ cells were cultivated for 24 h with 2-ME or with DMSO before CREB phosphorylation and CREB protein expression was determined by Western blot. 50 μg total protein per lane were used. The blot represents one of two independent experiments. **(C)** Binding of CREB to the CRE element was determined by EMSA analysis. Nuclear extracts of HER-2/neu^+^ cells were treated with DMSO or the indicated concentration of 2-ME prior to an EMSA reaction by incubating a biotinylated CRE oligonucleotide with the nuclear extract in reaction buffer. Following gel electrophoresis, the complexes were blotted onto a nylon membrane and DNA was detected with streptavidin-HRP. The diagram represents the densitometry of the shifted CREB-CRE complex obtained from two independent experiments. **(D)** The morphology of the 2-ME-treated cells was documented. The bar represents 50 μm. **(E)** Cell proliferation was measured by a BrdU ELISA in 96 well plate formats. Cells were incubated with BrdU and 2-ME or DMSO as the untreated control, respectively, for 24 h and the integrated BrdU was detected with a specific antibody after cell fixation. Columns represent the mean of three independent experiments with two technical replicates. The data were normalized to the DMSO-treated NIH3T3 control, which was set to 100%. **(F)** Metabolic or mitochondrial activity of the cells was measured by adding XTT reagent for 4 h to the cells after 24 h incubation period with the inhibitors. The absorption was determined with an ELISA reader. Columns represent mean values of three independent experiments with three technical replicates each. The data of all columns were normalized to the DMSO-treated NIH3T3 control, which was set to 100%.

Since hypoxia (1% O_2_) can increase the phosphorylation of CREB at serine residue 133 [32], it was analyzed whether hypoxia-related TFs, like HIF-1α, can influence the CREB activity. Therefore, HER-2/neu^+^ cells were incubated with the HIF-1α inhibitor 2-methoxyestradiol (2-ME) for 24 h resulting in a decreased HIF-1α protein expression (Figure 3A), which was linked with a down-regulation of CREB phosphorylation at serine 133 in HER-2/neu^+^ cells. This effect was already visible upon treatment of HER-2/neu^+^ cells with 1 μM 2-ME and was not further enhanced in the presence of higher 2-ME concentrations (Figure 3B). In contrast, NIH3T3 cells exhibited the highest CREB phosphorylation at 1 μM 2-ME, which was strongly reduced with higher concentrations of this substance. Furthermore, electromobility shift assays (EMSA) revealed that 2-ME inhibited the formation of CREB-CRE complexes by decreasing the phosphorylation of CREB (Figure 3C).

In addition, blocking HIF-1α changed the morphology of NIH3T3 from a fibroblast-like to a foci-like grown pattern, while HER-2/neu^+^ cells lost their ability to form bigger foci (Figure 3D). It is noteworthy that the treatment of both cell lines with 10 μM 2-ME did not altered the cell vitality significantly (data not show), but decreased concentration-dependent the proliferation (Figure 3E) and the metabolic / glycolytic activity (Figure 3F).

### CREB-mediated control of apoptosis in HER-2/neu^+^ cells

**Figure 4:**
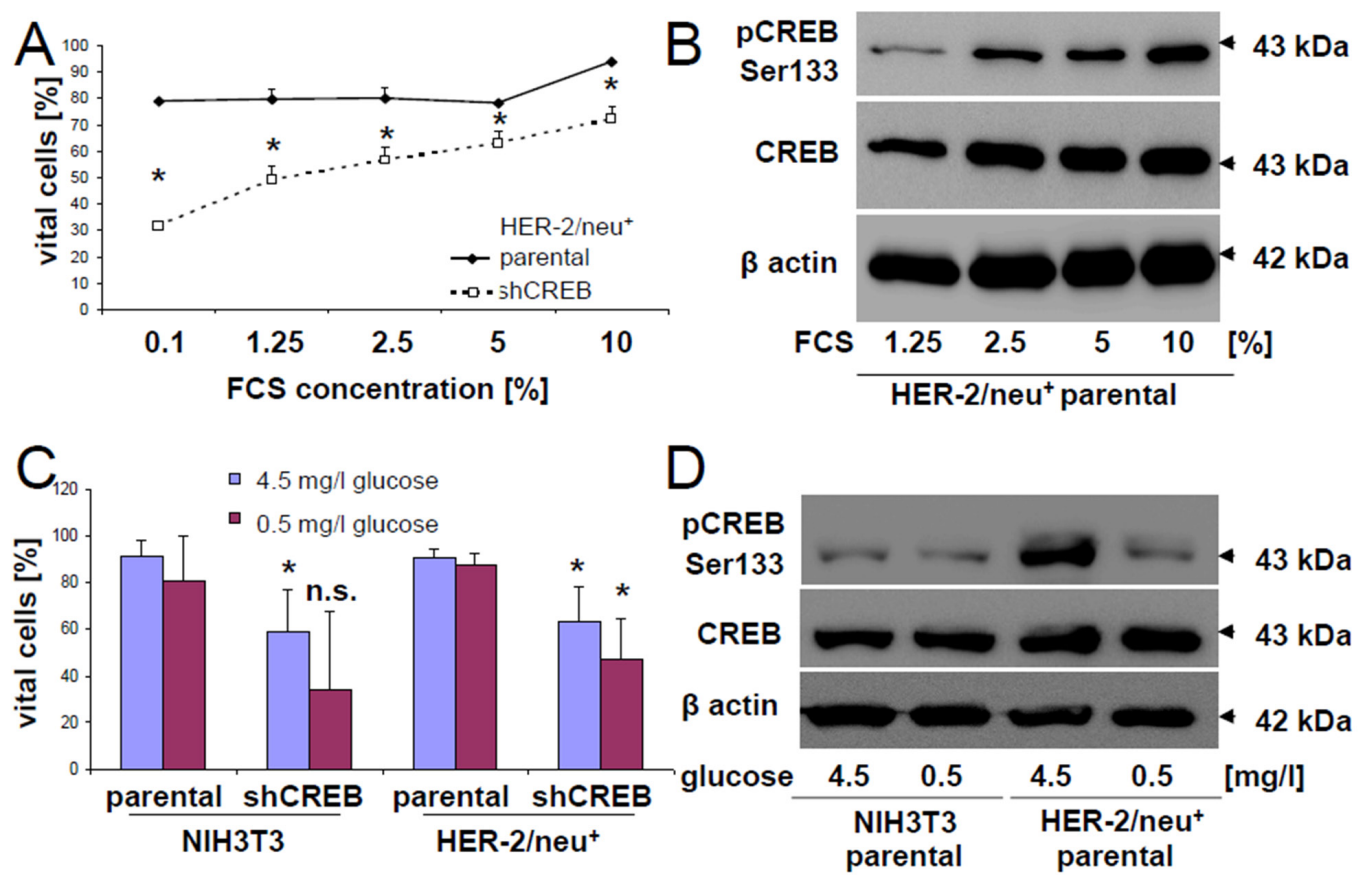
Regulation of the phosphorylation rate of CREB by glucose and FCS **(A)** HER-2/neu^+^ and shCREB derivatives were cultivated in EMEM supplemented with different FCS concentrations, before apoptosis was measured by flow cytometry after annexin V/PI staining as described in Materials and Methods. 5000 cells were measured per histogram and the experiment was independently repeated two additional times. **(B)** HER-2/neu^+^ cells were incubated with the indicated concentrations of FCS and the corresponding phosphorylation rate of CREB (upper panel) and of total CREB (middle panel) were determined by immune blotting whereas the detection of β-actin served as loading control. The blot represents one of two experiments. **(C)** HER-2/neu^+^ and shCREB derivatives were cultivated in RPMI with high (4.5 g/l) or low (0.5 g/l) glucose concentrations. The glucose content after addition of 10 % FCS was verified by DNSA measurement. The apoptosis rate of the cells after 24 h cultivation was quantified as described in (A). Data are the mean value and error bars from three experiments. **(D)** NIH3T3 and HER-2/neu^+^ cells were cultivated under low and high concentrations of glucose, before the corresponding phosphorylation rate of CREB was analyzed by Western blot as described in Materials and Methods. The blot represents one of two experiments.

In order to determine whether HER-2/neu^+^ cells are more resistant to apoptosis compared to shCREB cells, cells were cultured in medium supplemented with different FCS and glucose concentrations. CREB down-regulated HER-2/neu^+^ cells, but not the parental HER-2/neu^+^ cells cultured in 0.1 % FCS showed a reduced cell vitality as determined by flow cytometry upon annexin V / PI stainings (Figure 4A). Cultivation of HER-2/neu^+^ cells with increasing FCS concentrations correlated with higher CREB and pCREB levels, which were further correlated with the higher frequency rates of vital cells (Figure 4B).

In addition, glucose deprivation resulted in reduced cell vitalities in particular in CREB down-regulated HER-2/neu^+^ cells. The increased apoptosis rates were linked to reduced pCREB levels (Figure 4C) suggesting that growth properties, in particular the neoplastic features of HER-2/neu^+^ cells, are tightly linked with cell vitality. Moreover, supplementation of high glucose concentrations caused a significant induction of the corresponding pCREB levels (Figure 4D).

### Influence of altered CREB expression levels on the cellular metabolism

**Figure 5:**
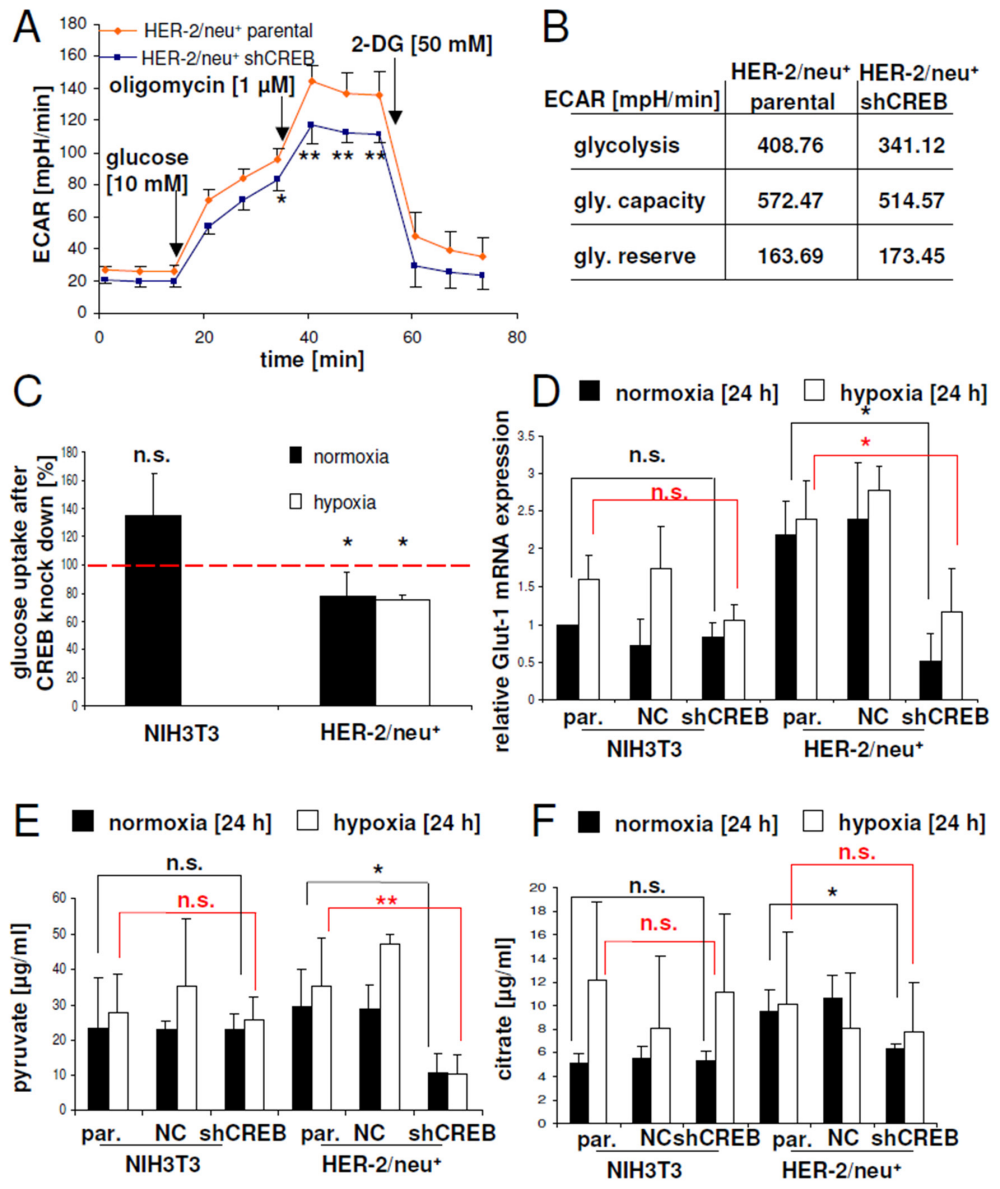
CREB-mediated regulation of glucose uptake by Glut-1 expression and glycolysis **(A)** Glycolytic activity was measured by the extracellular acidification rate. At the indicated time points glucose, oligomycin or 2-desoxyglucose (2-DG) was added. Data represents four independent experiments with eight replicates. **(B)** Glycolytic capacity and reserve were calculated with the data from (A). **(C)** Glucose uptake in NIH3T3 and HER-2/neu^+^ cells as well as their shCREB derivatives was analysed under normoxic and hypoxic conditions as described in Materials and Methods. Cells were seeded into 96 well plates with black walls in EMEM and were cultivated for 20 h under normoxic or hypoxic conditions. The cells were washed twice with PBS and 0.1 mM 2-NBDG was added. After 20 min. the cells were washed again with PBS and the plate was measured in a fluorescence ELISA at 485/525 nm. The experiment was repeated two more times. **(D)** The Glut-1 expression in shCREB transfectants and parental cell lines was determined by qPCR. The results are expressed as bar charts with mean and standard error from three independent experiments. The statistical analysis (between parental and shCREB cell lines) in black is from the normoxic dataset, while the red symbols are from the hypoxic dataset. Expression data were normalized to the cell line NIH3T3, which was set to “1”. **(E**, **F)** The concentrations of pyruvate (E) and citrate (F) in cell lysates under normoxia and hypoxia were measured with colorimetrical tests. The results are expressed as bar charts with mean and standard error from four independent experiments with two wells each. The statistical analysis (between parental and shCREB cell lines) in black is from the normoxic dataset, while the red symbols are from the hypoxic dataset.

Based on the results of the proteomics profiling and glucose deprivation experiments alterations within the glycolytic pathway in HER-2/neu^+^ cells vs. CREB down-regulated HER-2/neu^+^ cells are expected. Therefore, modulations of glycolysis pathways were determined by the extracellular acidification rates (ECAR). CREB down-regulation in HER-2/neu^+^ cells led to a marked decrease of the ECAR under baseline conditions as well as induction of glycolysis by adding glucose (Figure 5A). The glycolytic capacity of the cells, meaning the highest glycolytic activity under inhibition of the mitochondrial ATPase with oligomycin was lower in CREB knock down cells. This was accompanied with a diminished glycolytic activity observed in these cells (Figure 5B). In line with this observation, a reduced glucose uptake rate was recorded in CREB down-regulated HER-2/neu^+^ cells (Figure 5C), which was further associated with significantly decreased expression levels of the glucose transporter GLUT1 (Figure 5D) as well as with diminished pyruvate and citrate concentrations (Figure 5E, 5F). Interestingly, the concentrations of both glycolytic metabolites were reduced in the oncogenic HER-2/neu^+^ cell line after CREB knock down, but not in the parental NIH3T3 after diminishing of CREB by shRNA (Figure 5E, 5F). Under hypoxia the loss of pyruvate was even more pronounced in HER-2/neu^+^ shCREB cells, while it was unchanged in NIH3T3 shCREB cells.

**Figure 6:**
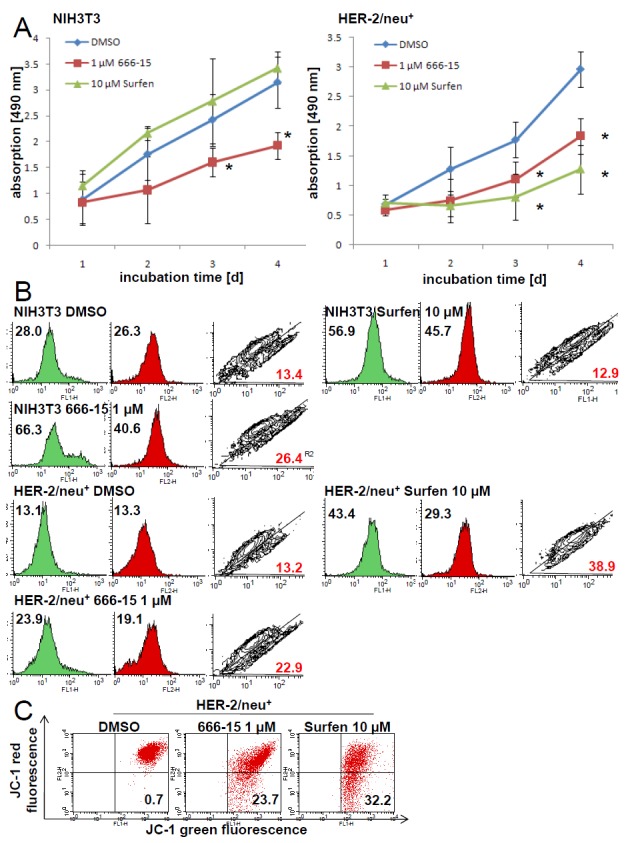
Inhibition of the mitochondrial activity and increased amount of dysfunctional mitochondria by CREB inhibitors 666-15 and surfen **(A)** NIH3T3 and HER-2/neu^+^ cells were incubated with 1 μM 666-15, 10 μM surfen or DMSO up to four days. For each time point XTT reagent was added for four h to the cell culture medium and cells were incubated at 37°C. Absorption was determined in 96 well plates with an ELISA reader. Data represents three independent experiments with three wells each. **(B)** The effect of the CREB inhibitors on “mitochondrial mass” and the mitochondrial activity was measured by staining the cultivated cells (24 h incubation period with the inhibitors) with 100 nM MitoTracker green and 100 nM MitoTracker red for 30 min following flow cytometrical analysis. 5000 events are displayed in every histogram and the mean fluorescence value is given in the histogram. Green areas are the MitoTracker green signals and the red filled histograms are the MitoTracker red signals. The contour plot shows the cells with dysfunctional mitochondria (R2) and the red number is the percentage of these cells with more mitochondrial “mass” (x axis) than mitochondrial activity (y axis). One out of three independent dataset is shown. **(C)** HER-2/neu^+^ cells treated with 666-15 or surfen for 24 h were stained with the dye JC-1 for 20 min at 37°C and the complex formation was analyzed by flow cytometry. CCCP was used as a positive control to set the quadrants. The number in the lower right quadrant is the amount of cells with a decreased mitochondrial potential. Histograms are from one of two independent experiments.

To validate if the loss of CREB binding activity has an effect on the cellular metabolism, two CREB inhibitors were used: 666-15, a potent inhibitor of CREB-regulated gene transcription [38], which disturbs the interaction of CREB and the CREB binding protein (CBP) resulting also in *in vivo* anticancer effects, and surfen, affecting the CREB-CRE complex [17, 39]. As determined by EMSA surfen prevented the formation of the CREB-CRE complex, while 666-15 had no effect on this interaction (Supplementary Figure 3A). Furthermore, surfen cannot displace ethidium bromide from the CRE oligonucleotide in a cell-free assay system (Supplementary Figure 3B) indicating that surfen did not bind to the CRE DNA element, but could interact with the basic leucine zipper of CREB. Both inhibitors diminished the proliferation by slowing down the cell cycle transition from the G1 to the S phase (Supplementary Figure 3C). This effect was more pronounced upon treatment with 666-15 than with surfen. Both 666-15 and surfen lowered the metabolic activity of HER-2/neu^+^ cells, while 666-15 but not surfen diminished the metabolic activity of NIH3T3 cells (Figure 6A).

Using specific fluorescence dyes we could show that 666-15 treatment resulted in an accumulation of dysfunctional mitochondria in HER-2/neu^+^ and NIH3T3 cells, while for surfen treatment this effect was only detectable in the HER-2/neu-transformed cell line (Figure 6B). This accumulation of dysfunctional mitochondria can be explained by the CREB-regulated “master switch for mitochondrial biogenesis” peroxisome proliferator-activated receptor gamma coactivator 1α [32] or by the loss of autophagy-related proteins ATG5 and ATG7 (Supplementary Figure 4A), a mechanism that can prevent mitochondrial dysfunctions in several cell lines [40, 41]. Furthermore, under normoxia and hypoxia an increased intracellular lactate level was determined in HER-2/neu^+^ shCREB cells (Supplementary Figure 4B). This can be caused by dysfunctional mitochondria and leading to an acidosis. Since pyruvate and citrate levels were decreased upon CREB knock down, we analyzed the levels of the metabolite acetyl-CoA. In both tested cell lines the concentration of acetyl-CoA was was increased when compared to the parental and to the vector control (Supplementary Figure 4C).

Since a loss of mitochondrial membrane potential was detected under treatment with both inhibitors (Figure 6C), the release of cytochrome c and ROS into the cytoplasm is suggested.

### Detoxification mechanisms regulated by CREB

**Figure 7:**
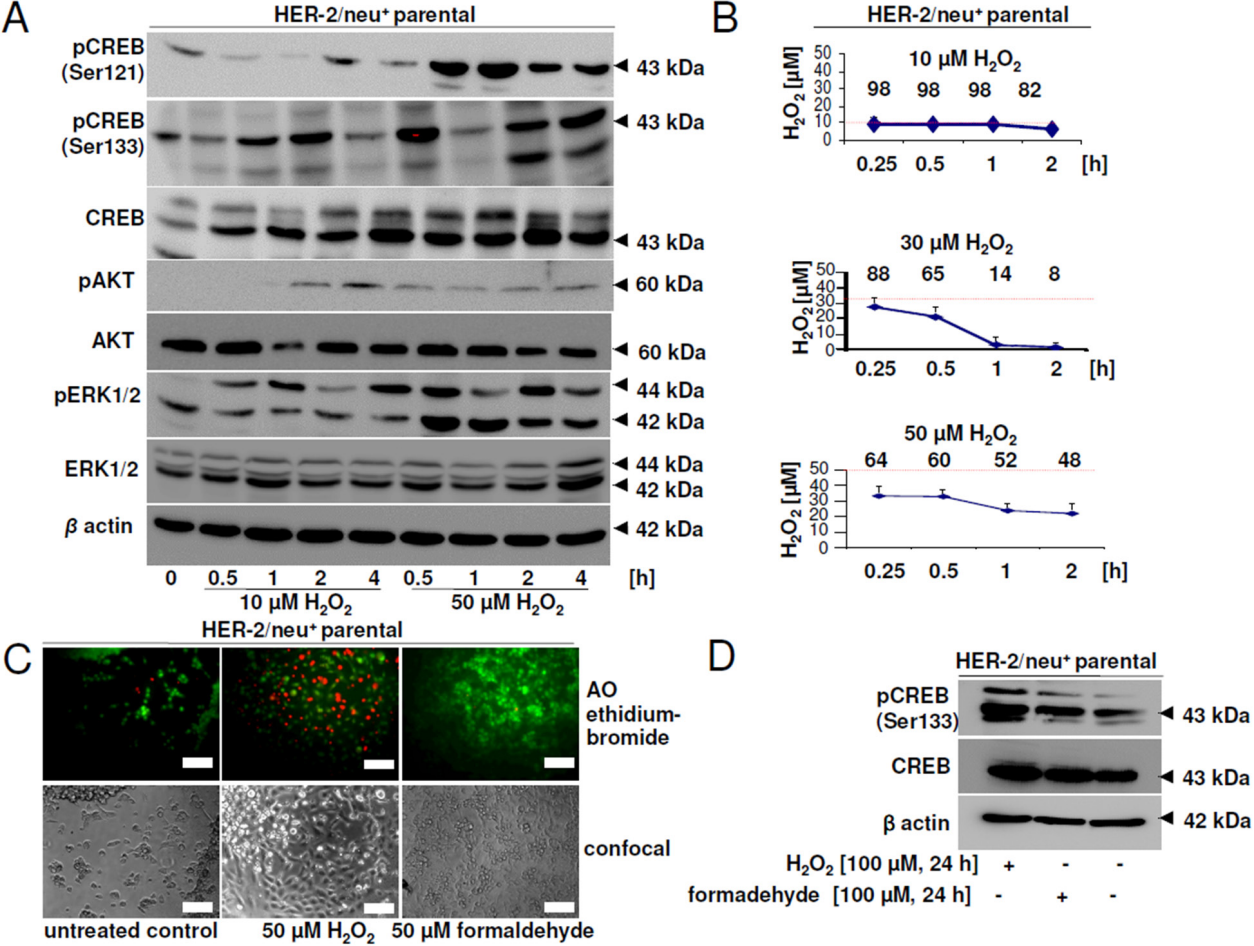
Effect of hydrogen peroxide and formaldehyde on CREB activity and viability **(A)** HER-2/neu^+^ cells were incubated with 10 or 50 μM H_2_O_2_ for the indicated time and phosphorylation of CREB, AKT and ERK was detected by Western blotting. Staining with an anti-β-actin mAb served as a loading control. The picture shows one of two independent Western blots. **(B)** Hydrogen peroxide in the cell culture supernatant was measured with the xylenol orange method and was compared to a standard curve. The red line indicates the starting concentration of H_2_O_2_ and the numbers are the remaining % of H_2_O_2_ at each time point. Data are the mean values from two independent experiments. **(C)** 80 % confluent cells were incubated with H_2_O_2_ or formaldehyde for 24 h and the dead cells were stained by adding 10 μM acridin orange and 10 μM ethidium bromide after treatment. Cells were visualized with a fluorescence microscope. The bar represents 50 μm. **(D)** Phosphorylation of CREB protein after long-term incubation with H_2_O_2_ or formaldehyde (24 h) was detected by specific antibodies as described in Materials and Methods. The experiment was performed three times and the blot is one representative picture.

One of the CREB-regulated proteins identified to be differentially expressed upon CREB down-regulation was catalase, an enzyme involved in the detoxification of hydrogen peroxide. Since oxidative stress can increase CREB phosphorylation and activity, the effect of different concentrations of H_2_O_2_ on HER-2/neu^+^ cells was determined. While low concentrations (10 μM) increased the CREB phosphorylation rate at Ser133, but not at Ser121 during 4 h, high concentrations of 50 μM H_2_O_2_ significantly increased CREB phosphorylation after 30 min. to 4 h (Figure 7A). The phosphorylation rate of AKT was strongly increased in response to exposure to 50 μM H_2_O_2_, while the phosphorylation level of ERK reached the maximal signal after a 4 h incubation period.

The most efficient detoxification of H_2_O_2_ was determined in the presence of 30 μM H_2_O_2_. Under these conditions almost 90 % of H_2_O_2_ was degraded after 2 h, while at higher or lower concentrations more than 50 % of the starting H_2_O_2_ levels remained still detectable in the given culture supernatants (Figure 7B). Exposure to 50 μM H_2_O_2_ significantly induced necrosis after 24 h (Figure 7C), which was absent at lower H_2_O_2_ concentrations (data not shown). Furthermore, incubation with formaldehyde over the same time had no effect on the vitality of the cells. However, long-term intervals (24 h) exposure resulted in increased phosphorylation rates of CREB (Figure 7D) regardless of the type of toxic compound (H_2_O_2_, formaldehyde).

**Figure 8:**
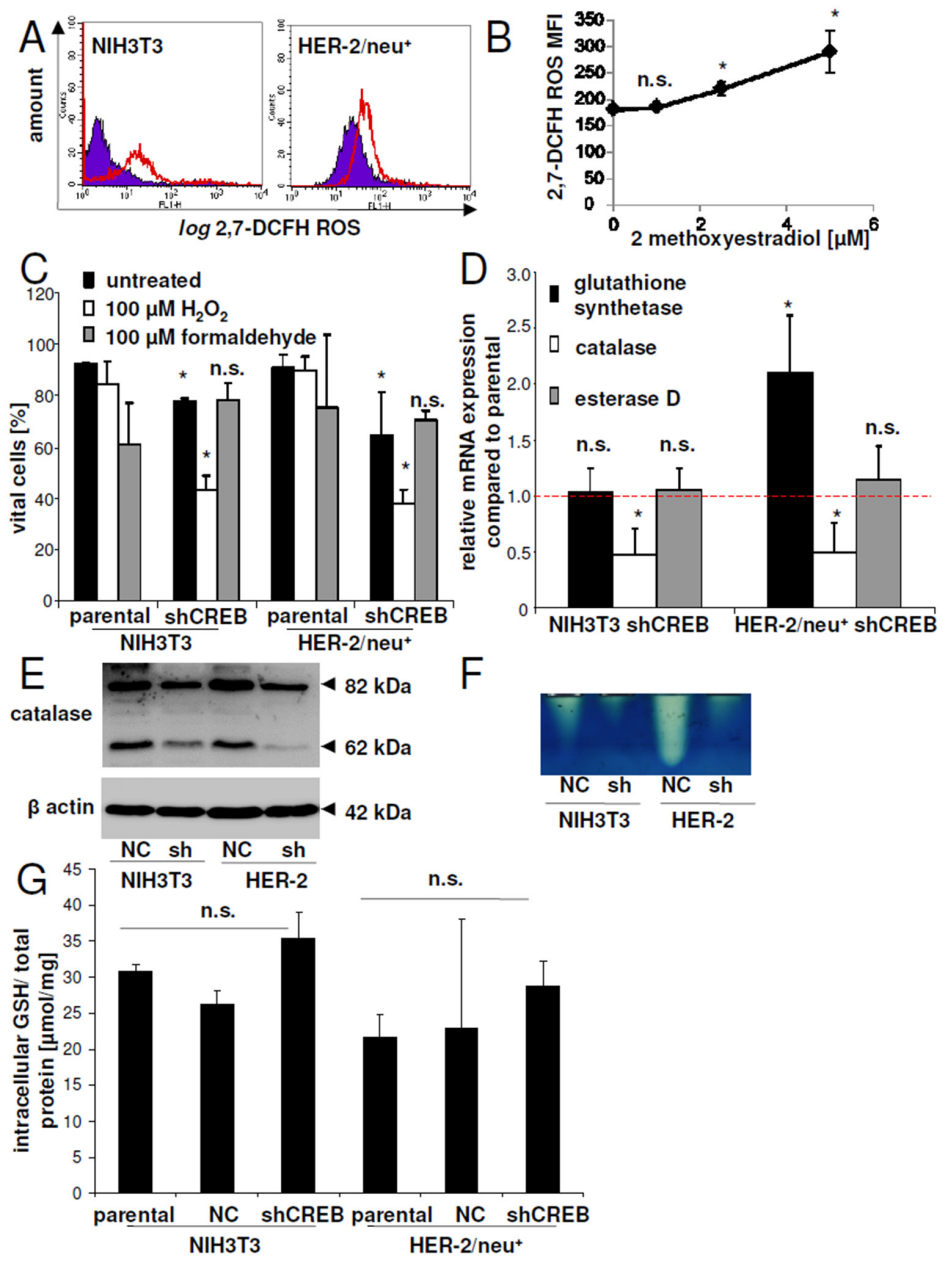
Regulation of detoxification enzymes by CREB **(A)** The ROS status/cell was determined by flow cytometry after staining with the ROS sensitive dye 2,7-DCFH. 10,000 cells were measured for one replicate. shCREB cells are within the red bordered area, while parental cells (NIH3T3 or HER-2/neu^+^ cells) can be found in the violet area. Representative histograms from one of three experiments are shown. **(B)** The mean 2,7-DCFH fluorescence intensity values of 2-ME treated cells was measured as in (A). The data points represents three independent experiments. **(C)** Viability of NIH3T3, HER-2/neu^+^ and their CREB down-regulated counterparts treated with high concentrations of H_2_O_2_ or formaldehyde for 2 h was measured with annexin V and PI staining. After staining 10,000 cells were analyzed by flow cytometry and cell viability was compared to that of untreated cells. Columns represens mean values of three independent experiments. **(D)** mRNA expression of catalase, esterase D and glutathione synthetase was determined in the cell lines. The red dashed line indicates the mRNA expression in the parental cell lines (NIH3T3 or HER-2/neu^+^), while the columns represent the mRNA expression in the CREB down-regulated cells. **(E)** Protein expression in NIH3T3 control cells (NC) and CREB down-regulated cells (shCREB) as well as in HER-2/neu^+^ cells was determined by Western blot using catalase specific antibodies. The Western blot is representative from one out of three experiments. **(F)** Cell lysates were separated on native gels and catalase activity was visualized by a catalase specific staining. The picture shows one experiment and was repeated thrice. **(G)** GSH concentrations in the cell lysates were determined using the glutathione assay kit as described in Materials and Methods. The concentration was normalized to the protein concentration. Data represents mean values of three independent experiments with two replicates.

Using the ROS sensitive dye 2,7-DCFH an accumulation of ROS in CREB down-regulated cells and in HER-2/neu^+^ transformants was detected, which was higher than that of parental NIH3T3 cells (Figure 8A). In the presence of the HIF-1α inhibitor 2-ME an accumulation of ROS was also found in HER-2/neu^+^ cells (Figure 8B). CREB knock down decreased the cell viability in the presence of H_2_O_2_ but not of formaldehyde-treated cells up to 60 % when compared to untreated cells (Figure 8C). We therefore analyzed the mRNA expression of three “detoxifying” enzymes important in cellular homeostasis: catalase, esterase D, an enzyme an enzyme involved in the detoxification of formaldehyde and glutathione synthetase, which is an important antioxidant. Only catalase mRNA expression levels were decreased in response to CREB down-regulation, while the mRNA expression levels of esterase D was not altered (Figure 8D). The decreased mRNA expression levels of catalase was accompanied by diminished catalase protein expression (Figure 8E) and consequently by a lower catalase activity (Figure 8F). In addition, glutathione synthetase was 2.1-fold up-regulated in CREB down-regulated HER-2/neu^+^ cells, but not in the parental NIH3T3 cells. However, free glutathione remained unaltered in all cell lines analyzed (Figure 8G).

### Disturbation of glycolysis and mitochondrial functions by ROS

**Figure 9:**
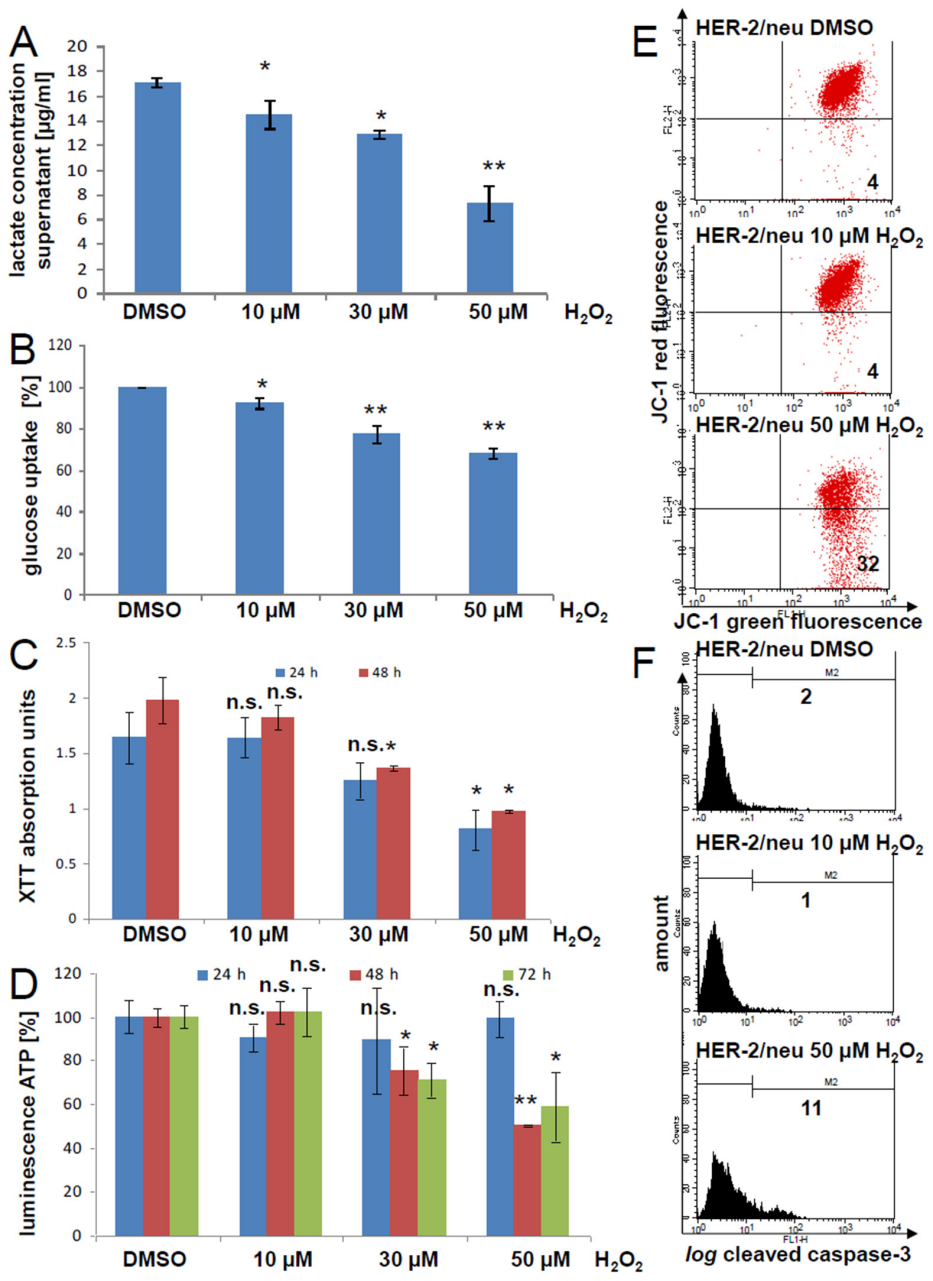
Inhibition of the metabolic and mitochondrial activities by hydrogen peroxide in HER-2/neu^+^ cells **(A)** The lactate concentration in the cell culture supernatant of HER-2/neu^+^ cells incubated for 24 h with the indicated H_2_O_2_ concentration was measured with an enzyme assay kit. A lactate standard was used for the calibration. Data represents two independent experiments with three replicates per run. **(B)** Glucose uptake was determined fluorimetrically by cultivation of H_2_O_2_-treated cells for 15 min with 1 mg/ml 2-NDBG. Fluorescence units were normalized to the DMSO control. Columns represent three replicates with three wells each. **(C)** HER-2/neu^+^ cells were incubated with the indicated concentration of H_2_O_2_ for 24 or 48 h and the mitochondrial activity was measured with XTT. Data represents two experiments with three replicates. **(D)** HER-2/neu^+^ cells were incubated with the indicated concentration of H_2_O_2_ for 24, 48 or 72 h and ATP concentrations was analyzed by using the “CellTiter glo” system. Data represents two experiments with three replicates. **(E)** The effect of 10 and 50 M H_2_O_2_ for a 24 h cultivation period on the mitochondrial potential was determined by JC-1 staining and fluorescence analysis. The number in the lower right quadrant is the amount of cells with damaged mitochondrial membranes. 5,000 cells were measured for every dot plot. The histograms show one of three independent experiments. **(F)** Cleavage of the effector caspase-3 was monitored by flow cytometry by using a cleavage specific antibody. 5,000 cells / sample were determined. The histogram is representative and the experiment was repeated twice. M2 represents the cells with an activated caspase-3 and the percentage is given.

Next, we tested the effect of H_2_O_2_ as a ROS on the glycolysis and the mitochondrial activity. In the culture supernatant of H_2_O_2_-treated cells lower concentrations of lactate was detectable than in the DMSO control (Figure 9A). Furthermore, H_2_O_2_ reduced the uptake of glucose as determined using the glucose analogue 2-NBDG (Figure 9B). Both mechanisms were concentration dependent. This led to lower metabolic activity (Figure 9C) and reduced ATP production (Figure 9D), which was not only due to a decreased cell proliferation (Supplementary Figure 5). However, higher concentrations of H_2_O_2_ decreased mitochondrial membrane potential (Figure 9E), as well as induced the cleavage of caspase-3 (Figure 9F).

## DISCUSSION

Major advances have been made in our understanding of the molecular mechanism of CREB function in malignant transformation and its association with hypoxia [17], but so far there exists only little information about the role of CREB in modulation of the cellular metabolism. Therefore, the aim of this study was to determine the effects of CREB and/or hypoxia-induced pathways on the cellular metabolism. Using 2-DE-based proteomic profiling of HER-2/neu^+^ versus HER-2/neu^+^ shCREB cells differentially expressed proteins were identified mainly involved in cell metabolism and localized in the cytoplasm. In particular, the expression of proteins associated with the glycolysis was altered. Validation of the CREB-regulated genes revealed in some cases a coordinated down-regulation of the respective mRNA and protein expression levels, while e.g. PGK 1 transcription remained unchanged upon CREB modulation, but altered at the protein level suggesting a posttranscriptional control. Thus, metabolic changes mediated by CREB seem to occur at both the transcriptional as well as at the posttranscriptional level. In this context it is also noteworthy that hypoxia could lead to posttranslational modifications of CREB [32]. The increased glycolysis of HER-2/neu^+^ cells was in accordance with the neoplastic properties of these cells. Different phosphorylation of CREB serine residues can affect metabolic enzymes, like PEPCK [42].

The altered metabolism due to HER-2/neu-controlled CREB overexpression modulates the TME. High lactate concentrations and low pH values were found in the supernatant of HER-2/neu^+^ cells, which might affect the anti-tumoral immune response. Indeed, acidic pH values could negatively interfere with the activity and function of effector T cells, dendritic cells as well as NK cells [43]. Furthermore, the direct influence of CREB on the cellular metabolism was strengthened by altered intra-cellular LDH levels. LDH representing the key enzyme of the glycolytic cascade and converting pyruvate to lactate is a CREB target [44]. Up-regulation of LDH levels led to an increased aerobic glycolysis, while CREB knock down not only reduced LDH levels in our HER-2/neu model system, but also in breast cancer [45]. The activation of the MEK/ERK pathway by HER-2/neu increased the CREB binding to the CREB-binding site (CRE) in the LDH promoter leading to its transcriptional up-regulation [46]. Furthermore, loss or reduced LDH expression abrogated cell proliferation *in vitro* and tumor formation *in vivo* in particular in triple negative mammary carcinoma [47], while high levels of LDH expression could be associated with poor patients’ outcome and inhibition of immune surveillance [48].

Comparative analysis of HER-2/neu^+^ and HER-2/neu^+^ shCREB cells demonstrated that CREB suppression correlated with an increased respiration and respiratory reserve. Thus, CREB-controlled altered metabolism was also associated with a reduced sensitivity to undergo apoptosis since HER-2/neu^+^ cells were able to grow under limited serum and glucose concentrations. This could be even further altered under hypoxic conditions leading to pronounced changes of the intracellular lactate dehydrogenase levels, extracellular lactate concentrations as well as extracellular pH values. *In silico* analysis of the transcriptional profile of chronic myeloid leukemia cells, in which CREB was knocked out demonstrated an upregulation of genes involved in tumor initiation and progression as well as in hypoxic signaling [49]. Since tumors with a higher glycolytic rate were associated with a worse prognosis accompanied by a poor clinical outcome of patients [48, 50], targeting of metabolic pathways might become an attractive therapeutic approach to treat cancer patients.

Increased ROS accumulation could be caused by mitochondrial dysfunction through the endoplasmic reticulum [51]. Furthermore, decreased mitochondrial activity implemented by mitochondrial dysfunction could activate CREB [52], which could in turn promote mitochondrial biogenesis [32]. Therefore, we tested whether oxidative stress like hydrogen peroxide could induce CREB activity *in vitro*. By upregulation of the AKT pathway the phosphorylation of CREB at Ser 133 was increased. Similar results were reported in a PKA-independent pathway by Pregni and co-authors [53] in neuronal cells, while treatment of melanoma cells with H_2_O_2_ increased CREB expression by the cAMP/PKA pathway [54]. Furthermore, H_2_O_2_ could stimulate phosphorylation of CREB by AKT thereby promoting mitochondrial respiration and biosynthesis [55], while the expression levels of esterase D (formyl glutathione hydrolase), an enzyme important for formaldehyde detoxification and upregulated by K-Ras mutations in murine fibroblasts [56], were not altered under these conditions.

**Figure 10:**
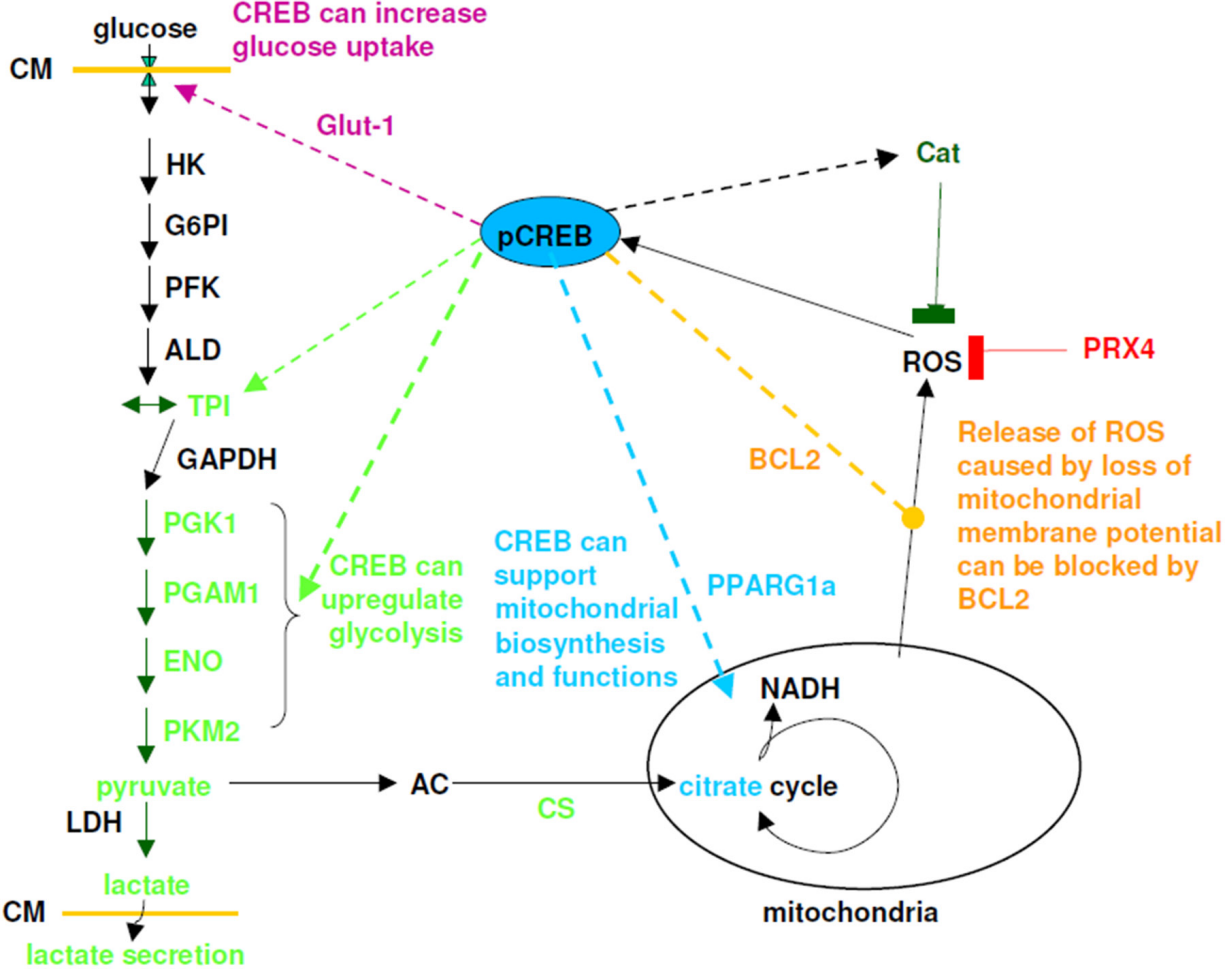
Model for effect of CREB on glycolysis, mitochondrial activity and detoxification mechanisms Solid lines are reactions or reaction pathways, while dashed lines indicates mechanisms regulated by CREB. Abbreviation used: CM = cytoplasma membrane, Glut1 = glucose transporter, HK = hexokinase, G6PI = glucose-6-phosphosphate isomerase, PFK = phospho-fructokinase, ALD = aldolase, TPI = triosephosphate isomerase, GAPDH = glycerin-3-phosphate dehydrogenase, PGK1 = phospho glycerate kinase, PGAM1 = phosphoglycerate mutase, ENO = enolase, PKM2 = pyruvate dehydrogenase, LDH = lactate dehydrogenase, AC = acetyl-CoA, CS = citrate synthase, PPARG1a = peroxisome proliferator activated receptor gamma, cat = catalase, PRX4 = peroxiredoxin 4, bcl2 = B-cell lymphoma 2.

CREB supports metabolism in several ways: First it can up-regulate the expression of Glut-1, therefore increasing uptake potential of glucose and intracellular glucose levels. Second, it can positive regulates some glycolytic enzymes (PKM2, TPI, alpha-enolase). Third it can enable mitochondrial respiration and biogenesis under stress conditions like hypoxia and ROS, therefore allowing a better survival of tumor and tumor cell (Figure 10).

However, further research is required for better understand the role of TFs, like CREB, in the mitochondria and their effect on the regulation of the cellular metabolism and oxidative stress under physiologic, but also pathophysiologic conditions [57].

## MATERIALS AND METHODS

### Cell culture, hypoxia, drug treatment and glucose uptake

The generation and culture conditions of HER-2/neu^-^ NIH3T3 cells, their HER-2/neu^+^ derivative and their respective CREB variants have been recently described [11, 34]. HER-2/neu-overexpressing cells with high levels of pCREB were termed HER-2/neu^+^ cells, HER-2/neu^+^ cells, in which CREB was down-regulated by shCREB were termed HER-2/neu^+^ shCREB cells.

For determination of the impact of FCS and glucose concentrations on the cell growth, cells were cultivated in 1 – 10 % FCS and 0.5 – 4.5 g/l glucose, respectively.

For hypoxia induction, cells were incubated in a 1 % (v/v) O_2_, 5% (v/v) CO_2_ atmosphere at 37°C for the indicated time spans [32]. Normoxic controls were cultured using standard conditions.

Cells were treated for 24 – 48 h with different inhibitors (HIF-1α inhibitor 2-methoxyestradiol, CREB-CBP inhibitor 666-15 (Tocris), CREB-CRE inhibitor surfen (Sigma), proton pump inhibitor Esomeprazole) and toxic components (H_2_O_2_, formaldehyde). Cytotoxicity of the substances was analyzed by treatment of cells with increasing concentrations of these inhibitors to determine the IC_50_ value by using the cytoto assay kit (Promega). Glucose uptake was determined as recently described in detail [58].

### RNA isolation, cDNA synthesis, qPCR

Total cellular RNA from 5 x 10^5^ cells/sample was extracted and subjected to qPCR analysis as recently described [11]. The specific primer sequences and PCR conditions used were provided in Supplementary Table 1. Relative expression levels were calculated according to the ΔCt method and normalized against β-actin and/or GAPDH as internal controls. Three independent experiments were performed.

### Protein isolation, western blot analysis and CREB ELISA

Total protein was isolated from 5 x 10^6^ cells/sample and the resulting lysates subjected to Western blot analysis as recently described [11] using the panel of antibodies listed in Supplementary Table 2. Blot membranes were stripped with 2.5 M glycine (pH 2) for 15 min. and were then re-probed with a new antibody after re-blocking with skim milk. For data analysis, three independent experiments were used.

The loss of CREB phosphorylation and protein was further quantified with a CREB specific ELISA system (Cayman). 10 μg total protein were incubated with the antibody coated plates for 1 h at 25°C before CREB phosphorylation and CREB protein expression were determined by adding the primary antibody. After three washing steps the samples were incubated with the HRP-linked secondary antibody for 30 min., washed three times and reaction was started by adding TNB substrate. After 30 min. the reaction was stopped with 1 mM acetic acid and absorption was measured af 490 nm. Three biological samples with three reactions were measured with each cell line.

### Flow cytometry

Cells were harvested after cultivation for 24 h under normoxia or hypoxia, respectively, and then stained for 20 min. with an anti-HER-2/neu-PE-conjugated (Becton Dickinson) antibody or with the IgG1-PE conjugated isotype control (BD), respectively, as recently described [59]. Fluorescence intensity was determined by flow cytometry on a FACSCalibur (BD). The results were expressed as mean specific fluorescence intensity (MFI) of three independent experiments.

For measuring the mitochondrial mass and the mitochondrial activity per single cell, the fluorescent dyes MitoTracker green (Cell signaling) staining the mitochondrial membrane, and MitoTracker red CFXRos (Invitrogen), which changed to a fluorescent dye under mitochondrial activity, were used. Cell pellets were resuspended in PBS supplemented with 100 nM of both dyes and then stained for 30 min. at 37°C in the dark. Cells were washed with PBS and directly analyzed by flow cytometry. Three biological replicates were used for every condition.

### Determination of apoptosis and caspase-3 activation

Annexin V - propidium iodide staining was used for the detection of apoptotic and necrotic cells as previously described [11]. For fluorescence analysis, 10 μM acridine orange and 10 μM ethidium bromide were added to the given cell culture. After a 10 min incubation period the samples were analyzed by fluorescence microscopy using both green / red filter settings. Three biological replicates with three biological replicated each were photographed.

To determine the caspase-3 activation through cleavage, the FITC Active Caspase-3 Apoptosis Kit (BD) was used following the manufacturer’s instruction. Briefly, 2.5 x 10^6^ cells were fixed in 500 μl cytofix/cytoperm at 4°C for 30 min., and then washed once with 1 ml cytowash, before they were stained in 100 μl cytowash and 10 μl anti-active-caspase-3-FITC for 30 min. After one additional washing step, 1x10^5^ cells/sample were analyzed on a FacsCalibur (BD). The experiment was independently repeated twice.

### Determination of H_2_O_2_ concentrations and enzyme activities

The concentration of H_2_O_2_ in the culture media was determined by the xylenol orange-Fe^2+^ method described by Gay and co-authors [60]. The enzymatic activity of PKM in cell lysates was measured by the method of Tietz and Ochoa [61]. 100 μl cell lysate were incubated with reaction puffer (50 mM Tris HCl pH 7.5, 4 mM MgCl_2_, 75 mM KCl, 0.2 mM ADP, 1.5 mM PEP, 0.2 mM NADH, 12 U/ml LDH) for 5 min. at room temperature. The reaction was started by addition of pyruvate kinase (end concentration 0.3 U/ml) and the absorbance change at 340 nm was recorded for 5 minutes.

Activity of prolyl endopeptidase was determined fluorometrically by the cleavage of the substrate Z-glycyl-prolyl-4-methylcoumarinyl-7-amide as described by Goossens and co-workers [62] with the following modifications: 100 μl of cell lysate were diluted with 100 μl incubation buffer (100 mM KH_2_PO_4_ pH 7.4, 100 μM NaN_3_, 1 mM DTT, 1 mM EDTA). Next the samples were incubated for 15 min. at 37°C and 10 μl of substrate was added (5 mM in DMSO). After incubation for 2 h in the dark at 37°C 500 μl 1.5 mM acetic acid were added and mixed. Fluorescence was analyzed with an ELISA reader (Tecan) with λ_ex_ 370 nm and λ_em_ 440 nm.

PGAM1 activity was measured following the protocol published by Hallows and co-authors [63].

TPI enzyme activity in cell lysate was analyzed by adding 2 ml reaction buffer (220 mM triethanolamine HCl, pH 7.6, 3 μM DL-glyceraldehyde-3-phosphate, 0.26 μM NADH, 50 mU glycerol-3-phosphate dehydrogenase, 0.2 U/ml triose phosphate isomerase) to 100 μl of cell lysate. The decrease in absorbance at 240 nm was recorded by a spectrometer.

PGK activity was determined by ATP formation: 100 μl cell lysate were mixed with 50 mM potassium phosphate, 5 mM 1,3-bisphosphoglycerate, 1 mM ADP, 5 mM CaCl_2_. The formation of Ca_3_(PO_4_)_2_ was measured by 340 nm after incubation by 25°C for 15 min.

For determination of the PRX 4 activity the protocol from Nelson and Parsonage [64] was used.

The enzymatic activity was normalizied to the total protein concentration in the cell lysate. All experiments were performed with three cell preparations.

### Extracellular flux assays

Bioenergetics of CREB down-regulated and CREB expressing HER-2/neu^+^ and HER-2/neu^-^ control cells were determined using the XF96e Extracellular Flux analyzer (Seahorse Bioscience) as recently described [65]. Briefly, 2x10^5^ cells/well were reseeded in specialized tissue culture plates (96FX micro well plate) and subsequently immobilized using CELL-TAK (BD Biosciences). One hour prior measurement, cells were incubated at 37°C in a CO_2_-free atmosphere. First, the basal extracellular acidification rate (ECAR), as an indicator for lactic acid production or glycolysis, was recorded. In the next step ECAR in response to the application of 1 μM oligomycin (XF Cell Mito Glyco Test Kit, Seahorse Bioscience) as well as 10 mM glucose was evaluated. All experiments were performed in four independent experiments with at least hexaplicates.

### Determination of lactate, pyruvate, citrate levels, acetyl-CoA and glucose concentrations in cell culture supernatants and cell lysats

The amount of lactate in 100 μl cell culture supernatants was analyzed with a lactate detection kit (Sigma) according to the manufacturer’s instructions in a 96 well plate format. The deproteinated samples were measured at 570 nm in an ELISA reader (Tecan) after enzymatic reaction. Three independent experiments were performed using three technical replicates in each run and the results were expressed as mean ± SD using bar charts.

For the detection of pyruvate and citrate in cell lysates commercial kits (Biovision) were used following the manufacturer’s protocol. Samples and standards were measured with an ELISA reader as described above.

Acetyl-CoA was measured fluorimetrically with an acetyl-CoA assay kit following the manufacturer’s instructions (Sigma).

The amount of glucose in the culture medium was controlled with the DNSA method as described by Miller [66].

### Proliferation and cell cycle analysis

Proliferation of treated cells was determined by measuring the DNA synthesis with the cell proliferation ELISA, BrdU (colorimetric) kit (Roche). 5 x 10^4^ cells were seeded per 96/well and were treated with or without H_2_O_2_. The inclusion of BrdU was analyzed following the manufacturer’s instructions. Three biological replicates with three biological sample each were analyzed at an ELISA reader (Tecan).

For cell cycle analysis the DNA content per cell was measured as described previously [11]. Briefly, after synchronization of the cells by serum deprivation (0.5% FCS) for 48 h, cells were incubated with media containing inhibitors and 10% FCS for the indicated time span. The cells were fixed after harvesting in 70% ethanol for at least 24 h by 4°C, following the isolation of nucleus by washing once with PBS, 0.5% tween 20 and then PBS, 0,5% tween 20 and 100 mM citrate. RNA was digested with RNase A treatment and DNA was stained with 0.1 mg/ml PI. DNA content was measured with a FacsAria III (BD) with three biological replicates for each condition.

### CREB – DNA binding analysis

Binding of CREB to the CRE DNA element was analyzed by an EMSA. Nuclear extracts of HER-2/neu^+^ cells were incubated with a biotinylated oligonucleotide (TGACGTCA) and the testing substances as described by Bukur and co-authors [59].

Furthermore, the ethidium bromide displacement assay was used to determine whether surfen interacts with the CRE element and therefore prevents binding of CREB or whether surfen binds to the CREB protein. The assay was conducted as published in Rishi and co-workers [67] for three times independently. The following hairpin oligonucleotids were used: TGACGTCAAAAAATGACGTCA (CREB), TGACTCAAAAAATGACTCA (AP1), TTAATTAAAAAA ATTAATTAA (AT rich), GGCCGGCCAAAAAGGCC GGCC (GC rich).

### Detection of reactive oxygen species (ROS)

Cells were incubated in 10 μM 2',7'-dichlorodihydrofluorescein diacetate (2,7-DCFH) for 15 min. at room temperature and then were washed twice with PBS. Fluorescence intensity was analysed by flow cytometry (BD) as recently described [68].

### Determination of glutathione levels

The glutathione levels were determined using the GSH/oxidized GSH ratio kit (Calbiochem) as previously described [56]. Briefly, 1 x 10^6^ cells were incubated for 48 h, washed twice with PBS, scratched of the plates and resuspended in 100 μl PBS. After cell lysis obtained by 6 cycles of freezing and thawing 900 μl 5 % (w/v) meta phosphoric acid/sample was added. 50 μl of the cell supernatant was analyzed according to the manufacturer’s instructions at 412 nm in a spectrometer (Merck). The amount of glutathione was correlated to the protein concentration determined with the bicinchoninic acid kit (Thermo).

### Catalase activity staining

Catalase staining was performed according to Weydert and Cullen [69]. 150 μg non-denaturated total protein lysate was separated with a native 8 % PAGE gel in a cold room (6°C) at ∼40 mA for 6 to 8 h. After electrophoresis the gel was washed with aqua dest. and incubated for 10 min by RT in 0.03 % H_2_O_2_. After washing twice with aqua dest. the gel was stained in 2 % (w/v) FeCl_3_ and 2 % (w/v) K_3_[Fe(CN)_6_]) until clear bands appeared. Finally, the gels were extensively washed with water and documented with a gel scanner. Four single protein lysates were used for every condition.

### Intracellular pH measurement

1 x 10^6^ cells were stained with 0.1 μM BCECF-AM for 30 min. at 4°C and washed twice with culture medium. 1 x 10^4^ cells/well were seeded into a black 96 well plate with a clear bottom and incubated at 37 °C for 24 h under normoxic or hypoxic conditions. The fluorescence intensity/well was determined in a fluorescence reader (Tecan, excitation wavelength 480 nm, emission wavelength 535 nm). For the analysis of pH stability and the regeneration time under normoxia, the fluorescence intensity was monitored for one h. For the standard curve the cells were incubated in buffer with known pH whereas nigericin-KCl (25 μM and 125 mM) was used for permeabilization.

### 2D gel electrophoresis, spot digestion and mass spectrometry

For determination of the differential protein expression pattern upon CREB down-regulation, 2-DE-based proteome analysis followed by mass spectrometry was performed as recently described [58]. Three biological replicates with four to six technical replicates (gels) were used for proteome analysis. Coomassie stained gels were documented with a gel scanner and gel pictures were compared with the Delta2D software (Decodon). The cut-off for up and down-regulation of protein spot was < 0.5 and > 2.5. Putative CREB-regulated proteins were cut-out with an automatic spot picker (Spothunter, Herolab) and prepared for MALDI TOF/TOF-MS with a α-cyano-hydroxycinnamic acid matrix. MS data were identified with the Mascot database. Protein spots with a score value higher than 55 and found in all three biological replicates were used for further validation processes.

### Statistical analysis

Differences between groups were analyzed by Mann-Whitney test, Student’s t test or ANOVA as appropriate. Data are expressed as mean ± SD and the corresponding *p* values reported are 2-sided. Significance was accepted if *p* values were ≤ 0.05 (*). The statistical analysis of the graphs and tables compares the CREB diminished cells with their parental cell lines (NIH3T3 or HER-2/neu^+^) if nothing else is stated in the figure legend. For the analysis of putative CRE regions in the listed gene promoter sequence the CREB target gene database (natural.salk.edu/CREB/) was used [70].

## SUPPLEMENTARY MATERIALS FIGURES AND TABLES



## Abbreviations

2-ME: 2-methoxyestradiol
2-NBDG: 2-[N-(7-nitrobenz-2-oxa-1,3-diazol-4-yl)amino]-2-deoxy-D-glucose
2-DE: two-dimensional gel electrophoresis
2,7-DCFH: 2',7'-dichlorodihydrofluorescein diacetate
ATCC: American Tissue Culture Collection
CaMK: calcium activated calmodulin kinase
CBP: CREB binding protein
CRE: cAMP-responsive element
CREB: cAMP-responsive element binding protein
ECAR: extracellular acidification rate
EMSA: electromobility shift assays
ENO: enolase
ERK: extracellular-signal regulated kinase
ESOM: Esomeprazole
FGF: fibroblast growth factor
GLUT: glucose transporter
GSH: glutathione
KID: kinase-inducible domain
KIX: KID-interacting domain
LDH: lactate dehydrogenase
mAb: monoclonal antibody
MALDI-TOF: matrix-assisted laser desorption/ionization time-of-flight
MAPK: mitogen-activated protein kinase
MCT: monocarboxylate transporter
MEK: MAP/ERK kinase
MFI: mean fluorescence intensity
NF: nuclear factor
OCR: oxygen consumption rate
P: *p*-value
PBS: phosphate-buffered saline
pCREB: phosphorylated CREB
PEP: prolyl endopeptidase
PGK: phosphoglycerate kinase
PGAM: phosphoglycerate mutase
PKA: protein kinase A
PKM: pyruvate kinase
PRX: peroxiredoxin
PTM: post-transcriptional modification
qPCR: quantitative PCR
ROS: reactive oxygen species
RTK: receptor tyrosine kinase
TF: transcription factor
TME: tumor microenvironment
TPI: triose phosphate isomerase
UTR: untranslated region
