# Supplementary material for: Linking CREB function with altered metabolism in murine fibroblast-based model cell lines
Source: Oncotarget. 2017 Oct 27;8(57):97439–63. doi: 10.18632/oncotarget.22135 (PMC5722575; doi:10.18632/oncotarget.22135)
Supplement: Supplementary file 1 [file oncotarget-08-97439-s001.pdf]

Linking CREB function with altered metabolism in murine fibroblast-based model cell lines

SUPPLEMENTARY MATERIALS

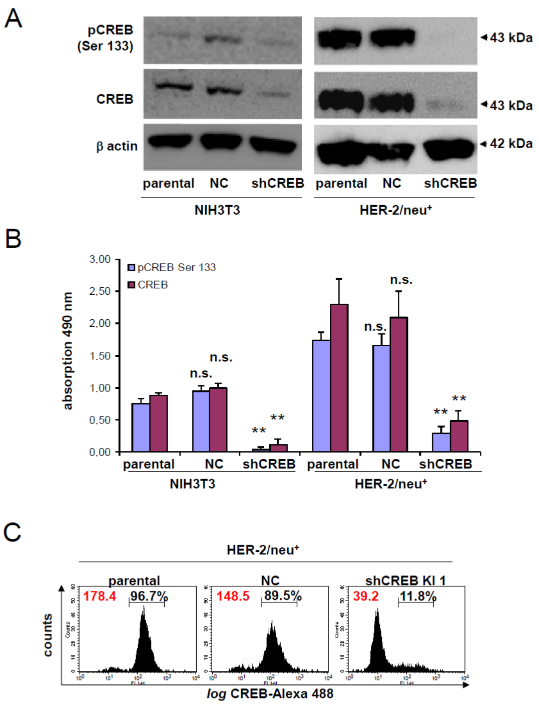

Supplementary Figure 1: CREB status of the murine fibroblast cells used. (A) Cell lysates of HER-2/neu^{+} overexpression murine fibroblasts (HER-2/neu^{+} parental), a cell line with a down-regulated CREB expression by shRNA constructs (shCREB) and a vector control (NC) were loaded onto a SDS gel and were blotted on a nitrocellulose membrane. CREB expression and phosphorylation were detected with specific antibodies. An anti-β-actin mAb served as a loading control. **(B)** Cell lysates were also used for the quantification of CREB phosphorylation and activity in the cell lines in (A) by using a commercial assay kit. Columns represent mean values from two independent experiments with four reactions. For the statistical analysis the values were compared to the parental cell line (NIH3T3 or HER-2/neu^{+}). **(C)** The individual CREB protein amount in the cell cultures was measured by flow cytometry. CREB protein was detected with an Alexa-488 secondary antibody, which binds to the primary CREB antibody. The histograms represent 5,000 cells and the mean fluorescence intensity (red number) and the % of cells in region given.

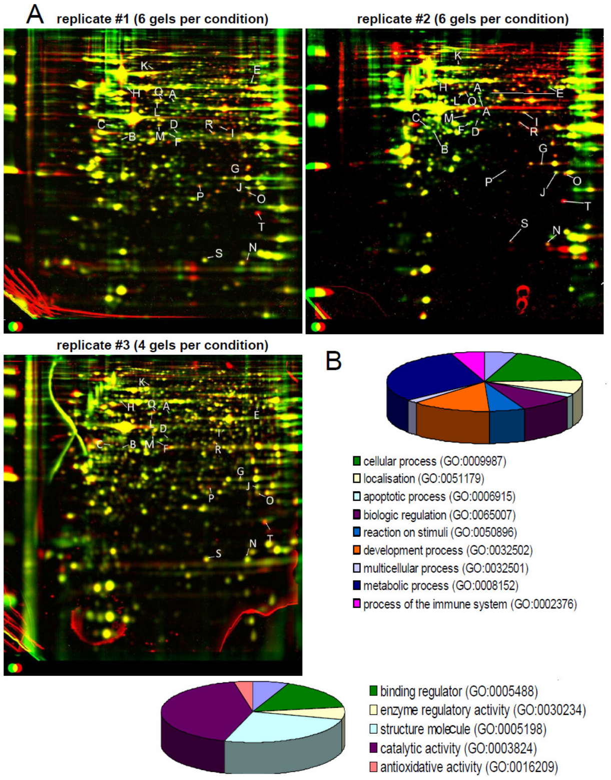

Supplementary Figure 2: Comparison of differentially regulated protein spot on the three merged 2-D gels. **(A)** The merged gels from HER-2/neu+ parental vs. HER-2/neu+ shCREB cells are shown. Red spots are up-regulated in HER-2/neu+ shCREB cells and green spots are down-regulated. Differentially expressed and identified protein spots are labeled in the subpanel: A = pyruvate kinase isozymes M1/M2, B = heat shock protein HSP 90-alpha, C = tubulin alpha-1A chain and isoforms 1B, 1C, 3 chain, D = phosphoglycerate kinase 1, E = catalase, F = alpha-enolase, G = phosphoglycerate mutase 1, H = vimentin, I = protein disulfide-isomerase A6, J = triosephosphate isomerase, K = prolyl endopeptidase, L = spliceosome RNA helicase Ddx39b, M = 26S proteasome non-ATPase regulatory subunit 13, N = superoxide dismutase [Cu-Zn], O = flavin reductase (NADPH), P = peroxiredoxin-4, Q = ATP-dependent RNA helicase DDX39A, R = leukocyte elastase inhibitor A, S = cofilin-1, T = alpha-crystallin B chain. **(B)** The biological functions (upper panel) and the molecular functions (lower panel) of the identified, differential regulated protein spots were determined with the PANTHER database (http://pantherdb.org/).

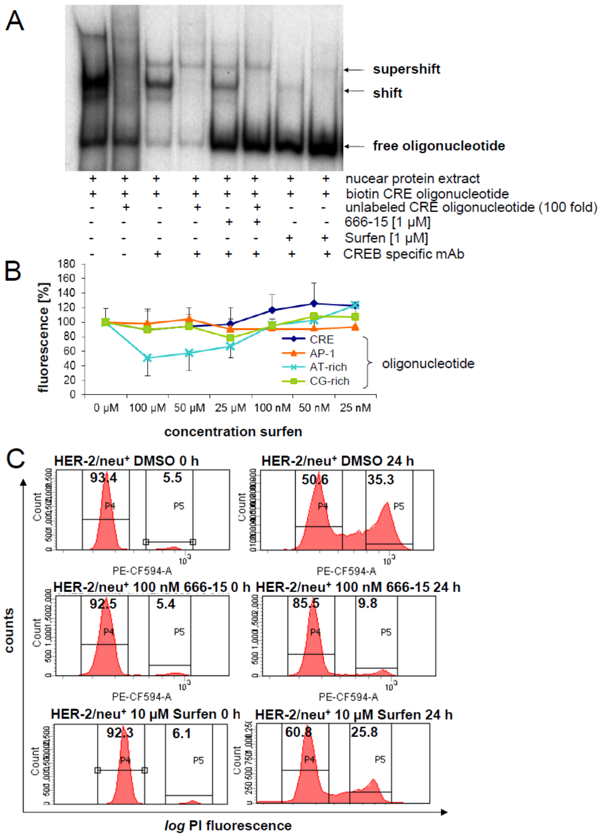

Supplementary Figure 3: Inhibition of the CREB – CRE complex formation by surfen prolonged cell cycle progression. **(A)** Nuclear extracts of HER-2/neu$^+$ cells were incubated in EMSA buffer with a biotinylated CRE oligo (TGACGTCA). In controls, an unlabeled CRE oligonucleotide was added. 666-15 and surfen were added in 1 µM concentrations each. A specific CREB antibody leads to a supershift of the CREB-CRE complex. The blot shows the representative results from one out of two experiments. **(B)** A hairpin oligonucleotide containing the sequence for CRE or AP-1, respectively, was incubated with ethidium bromide and different concentrations of surfen. The AT-rich and GC-rich oligonucleotides were used as a control. The fluorescence was determined with an ELISA reader and the fluorescense of the DMSO control was normalized to 100%. Data represents the mean value and error of three independent experiments and the statistic is given to the untreated control. **(C)** Cell cycle analysis of HER-2/neu$^+$ cells treated with 666-15 or surfen was performed by measuring the DNA content of the cells with PI. 10,000 cells were analyzed per sample.

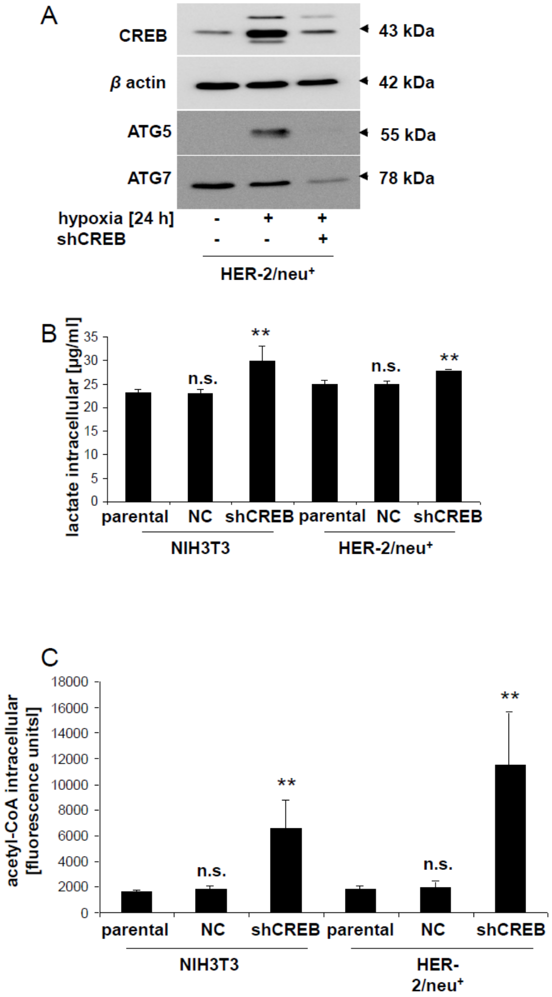

Supplementary Figure 4: Altered intracellular lactate levels and expression of autophagic markers by hypoxia. (A) The CREB, ATG5 and ATG7 protein expression of HER-2/neu^{+} cells under normoxia and hypoxia was compared with HER-2/neu^{+} shCREB cells under hypoxia. One of two experiments is shown. **(B)** 1 x 10^{6} cells incubated under normoxic and hypoxic conditions were used for a lactate assay. The bar charts are the mean value and error bars of two independent experiments with two technical replicates. Significance is given between the vector control and the parental (NIH3T3 and HER-2/neu^{+}) as well as shCREB and the parental (NIH3T3 and HER-2/neu^{+}). **(C)** Acetyl-CoA levels of 1x10^{6} cells were determined by using fluorescence measurements. Data represents three independent experiments.

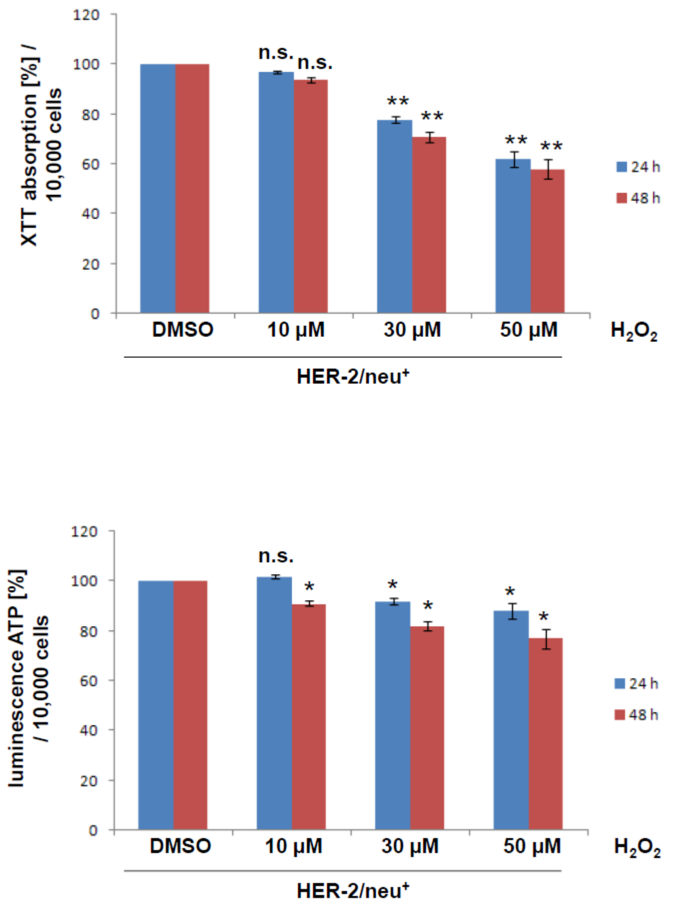

Supplementary Figure 5: H_2O_2-mediated loss of ATP production and metabolic activity. Cells were treated for 24 and 48 h with the indicated concentration of H_2O_2, harvested and 1 x 10^5 cells were used for the XTT (upper panel) or ATP (lower panel) analysis as described in Materials and Methods. Mean values and error bars from two independent experiments with three replicates are shown in the graphs. Significance of the values was compared to the untreated controls.

Supplementary Table 1: Primer used for real time quantitative PCR

Primer	Sequence	Annealing temperature [°C]
GAPDH fwd	TTGTGCAGTGCCAGCCTCGT	60
GAPDH rev	TCGGCCTTGACTGTGCCGTT	60
Catalase fwd	CAGTGCGCTGTAGATGTGAAA	60
Catalase rev	GTGTGAATTGCGTTCTTAGGC	60
Citrate synthase fwd	GTGACCATGAGGGTGGTAATG	60
Citrate synthase rev	CCGTCCTGAATTGAGTGTGTT	60
Cofilin 1 fwd	ACACCCCTACTCCGTATCCC	60
Cofilin 1 rev	CAGGGTCCCCAAAATCCCAA	60
alpha-crystallin, B chain fwd	ACACCCCTACTCCGTATCCC	60
alpha-crystallin, B chain rev	CAGGGTCCCCAAAATCCCAA	60
Esterase D fwd	ATTTGCTCCAATTTGCAACC	60
Esterase D rev	GGGAGTAACTGCCCATTTGA	60
Glucose transporter 1 fwd	TGTGCTGTGCTCATGACCATCGC	60
Glucose transporter 1 rev	AGCTCGGCCACAATGAACCATGG	60
Glutathion synthetase fwd	GCCTCCTACATCCTCATGGA	60
Glutathion synthetase rev	CCACATGCTTGTTCATCACC	60
Peroxiredoxin 4 fwd	AGGCTTGGAGAGTGATGAACG	60
Peroxiredoxin 4 rev	TTCGATCCCCAAAAGCGATGA	60
PGAM 1 fwd	TTGAAGCCCATCAAGCCCAT	60
PGAM 1 rev	GTAGGAGTCTGCCTCTTCGC	60
PGK 1 fwd	GGCATTCTGCACGCTTCAAA	60
PGK 1 rev	CGACATTTTGGCAACACCGT	60
PKM 1/2 fwd	CTGCAGGTGAAGGAGAAAGG	60
PKM 1/2 rev	GATGCAAACACCATGTCCAC	60
Prolyl endopeptidase fwd	TTTTCCGAGAGGTGACGGTG	60
Prolyl endopeptidase rev	TGGGAATCTTGGTGCCATCC	60
TPI 1 fwd	TCGGGGAGAAGCTAGACGAA	60
TPI 1 rev	TGAGCCACCCCATCATTGAC	60

Supplementary Table 2: Antibodies used for western blotting

Antigene	Animal	Manufacturer	Clone	Dilution	Dilution buffer
CREB-1	rabbit	Cell signaling	48H2	1:1000	5 % BSA, TBS-T
pCREB (Ser133)	rabbit	Cell signaling	87G3	1:1000	5 % BSA, TBS-T
pCREB (Ser121)	rabbit	Novus	B	1:1000	5 % BSA, TBS-T
beta actin	mouse	Sigma	AC74	1:5000	5 % SMP, TBS-T
AKT	rabbit	Cell signaling	polyclonal	1:2000	5 % BSA, TBS-T
pAKT Ser473	rabbit	Cell signaling	polyclonal	1:1000	5 % BSA, TBS-T
ATG5	rabbit	Cell signaling	D5F5U	1:1000	5 % BSA, TBS-T
ATG7	rabbit	Cell signaling	D12B11	1:1000	5 % BSA, TBS-T
catalase	rabbit	Biomol	n/a	1:2000	5 % BSA, TBS-T
α-Enolase-1	rabbit	GeneTex	polyclonal	1:2000	1 % SMP, TBS-T
ERK-1/2	rabbit	Cell signaling	polyclonal	1:2000	5 % BSA, TBS-T
pERK-1/2	rabbit	Cell signaling	D1314.4E	1:2000	5 % BSA, TBS-T
PDK-4	rabbit	Biorbyt	polyclonal	1:1000	5 % BSA, TBS-T
peroxiredoxin 4	rabbit	GeneTex	polyclonal	1:2000	1 % SMP, TBS-T
PGAM1	rabbit	Biorbyt	polyclonal	1:1000	5 % SMP, TBS-T
PGK-1	rabbit	GeneTex	polyclonal	1:1000	1 % SMP, TBS-T
PKM 1/2	rabbit	GeneTex	polyclonal	1:5000	5 % SMP, TBS-T
TPI-1	rabbit	GeneTex	polyclonal	1:2000	5 % SMP, TBS-T

BSA: bovine serum albumin; SMP: skim milk powder.
